# Evaluating the effect of probiotics on severe necrotising enterocolitis in preterm infants born before 32 weeks gestation in England and Wales: a propensity-matched population study

**DOI:** 10.1016/j.lanepe.2025.101571

**Published:** 2025-12-22

**Authors:** Alice Aveline, Lisa Szatkowski, Janet Berrington, Kate Costeloe, Shalini Ojha, Paul Fleming, Cheryl Battersby

**Affiliations:** a Neonatal Medicine, School of Public Health, Faculty of Medicine, Imperial College London, UK; b Centre for Paediatrics and Child Health, Imperial College London, London, UK; c Centre for Perinatal Research, Lifespan and Population Health, School of Medicine, University of Nottingham, UK; d Translational and Clinical Research Institute, Newcastle University, Newcastle upon Tyne, UK; e Centre for Genomics and Child Health, Queen Mary University of London, UK; f Homerton Healthcare NHS Foundation Trust, London, UK; g Neonatal Unit, University Hospitals of Derby and Burton NHS Trust, Derby, UK; h Department of Paediatrics, University College Dublin, Ireland; i Department of Neonatology, Newcastle Upon Tyne Hospitals NHS Foundation Trust, Newcastle upon Tyne, UK

**Keywords:** Probiotics, Preterm infants, Necrotising enterocolitis, Retrospective cohort studies, Population study, Propensity score, England, Wales, United Kingdom

## Abstract

**Background:**

Necrotising enterocolitis (NEC) remains an important cause of morbidity and mortality in preterm infants. This study aimed to examine whether probiotics reduce the risk of severe NEC and other key neonatal morbidities including late onset sepsis and mortality.

**Methods:**

Retrospective study using the United Kingdom National Neonatal Research Database. Infants <32 weeks gestation in England and Wales (01/01/2016–31/12/2022) were included if alive on day four, without major congenital anomaly. A propensity score matched approach was applied matching for gestational age cohorts, birth year epochs and 17 further items. Comparators were infants who were exposed or not to probiotics within 14 days. Primary outcome was severe NEC (confirmed at laparotomy or postmortem or listed primary cause of death). Parent focus groups and former NICU patients supported this study but did not contribute to design or writing.

**Findings:**

48,048 infants (45.2% (21,695/48,048) female), median gestational age 29.4 weeks (IQR 27.4–30.9) were included; 25.3% (12,161/48,048) were exposed to at least one of five available probiotics. 3.6% (1728/48,048) had severe NEC. Of 16,586 infants (8293 exposed and 8293 unexposed) in the propensity-matched analysis, incidence and odds ratios (OR) (95% CI) for exposed versus unexposed for severe NEC was 3.3% versus 4.2%, OR 0.80 (0.72–0.89); *other definitions of NEC yielded similar results.* Incidence for late onset sepsis (10.8% versus 11.5%, OR 0.94 (0.90–0.97)) and survival to discharge (96.6% versus 94.2%, OR 1.76 (1.65–1.88)). In infants <28 weeks gestation, severe NEC (8.7% versus 9.8%, OR 0.88 (0.82–0.93) and for ≥28 weeks (1.0% versus 1.7%, OR 0.59 (0.47–0.73).

**Interpretation:**

Probiotics were associated with a reduction in severe NEC including in those <28 weeks gestation. We currently recommend neonatal units not already using probiotics, to consider the introduction of products meeting appropriate recommendations, in the context of their local morbidity rates.

**Funding:**

NIHR Advanced Fellowship (CB reference: NIHR300617), Imperial College PhD studentship (AA), NIHR RfPB grant (NIHR203590, SO), Imperial NIHR Biomedical Research Centre.

**Research in context**

**Evidence before this study**

Some neonatal units use probiotics prophylactically to reduce the risk of necrotising enterocolitis, a common but serious complication of preterm birth. The last Cochrane meta-analysis included 10,918 infants from publications up to July 2022 and showed probiotics may reduce the risk of NEC (RR 0.54, 95% CI 0.46–0.65; I2 = 17%; 57 trials, low certainty). Using the Cochrane search strategy, we conducted a literature review in Embase and Medline (up to 25th July 2025) and identified an additional three relevant randomised control trials (RCT), with maximum sample size of 219, of which two trials showed benefit. Additionally, much observational data exists, with a 2022 meta-analysis of non-randomised data including 30 studies (n = 77,018) having an OR for NEC of 0.60 (95% CI: 0.50, 0.73; P < 0.00001). We found an additional three relevant observational studies, which included infants born <1000*g*, all showing benefit. Interpretation of the evidence is hampered by there being many statistically underpowered trials with limited, separately reported data for babies born before 28 weeks. Observational data in infants <1000*g* supports probiotic use. In addition, the optimal strains remain uncertain.

**Added value of this study**

This study adds real world UK data in a large population born <32 weeks between 2016 and 2022, where exposure to probiotics within the first 14 days was associated with a reduction in the risk of necrotising enterocolitis (across three definitions), late onset sepsis and mortality. The findings were consistent when logistic regression was applied to the whole cohort (48, 048 infants) and when a propensity-matching approach was applied to mitigate confounding (8923 matched pairs). These effects were observed in a subgroup analysis of infants born <28 weeks gestation and those born weighing <1000*g*.

**Implications of all the available evidence**

This large population study demonstrates important survival and morbidity benefits for preterm infants <28 weeks and <32 weeks gestation who receive probiotics. Findings are consistent with the Cochrane meta-analysis and show reductions in NEC, sepsis and mortality. We recommend neonatal units examine their own rates of NEC, LOS and mortality and consider implementing appropriate probiotic products.

## Introduction

Necrotising Enterocolitis (NEC) remains one of the most important causes of morbidity and mortality in preterm infants.^1^ In the United Kingdom (UK), the incidence of severe NEC (defined as NEC confirmed at laparotomy or postmortem or listed as the primary cause of death) was 3.2% among infants born <32 weeks gestation.^2^ The possibility that probiotics might safely reduce the risk of NEC has been investigated in randomised controlled trials (RCTs) and observational studies using different probiotic strains and combinations. However, the majority of studies were underpowered to investigate NEC as a primary outcome. In the most recent Cochrane meta-analysis,^3^ probiotics were shown to reduce the risk of NEC; probably reduce mortality; and have little or no effect on the risk of late-onset invasive infection. Trial data for extremely low birthweight (<1000*g*) or extremely preterm (<28 weeks gestation) infants were limited, and there was a recognition of the need for further trials.^3^ Whilst meta-analysis and contributing RCT data may be viewed as the gold standard, different authors use differing criteria for inclusion, and observational data can also contribute importantly to this debate.^4^^,^^5^

Recommendations from professional bodies vary. In 2020, the European Society for Paediatric Gastroenterology Hepatology and Nutrition (ESPGHAN), recommended that certain probiotics could be used as potential NEC reduction strategies.^6^ In 2021, the American Academy of Pediatrics issued a statement not supporting routine probiotic administration, citing a lack of evidence of benefit and limited availability of pharmaceutical grade products.^7^ More recently, following a neonatal case of probiotic sepsis, the US Federal Drug Administration (FDA) issued warning letters to manufacturers regarding the sale of unapproved probiotic products.^8^

In the UK, neonatal services are organised in geographically defined operational delivery networks (ODNs) and infants are transferred between neonatal units to receive appropriate care. We have previously reported the increase in probiotic use in England and Wales, from 9% of infants born <32 weeks in 2016 to 54% in 2022.^9^ Currently, of the five probiotic products in use over the study period, the two most commonly used probiotics in the UK are a combination of *Bifidobacterium infantis Bb-02, Streptococcus thermophilus TH-4* and *Bifidobacterium lactis BB-12* (marketed as Proprems, abbreviated as PP) and a product containing *Lactobacillus acidophilus NCFM*, *Bifidobacterium bifidum Bb-06* and *B. infantis Bi-26* (marketed as Labinic, abbreviated as LB).^9^

In this study, we harness the utility of large population datasets to assess the efficacy of probiotics used in contemporary, real-world practice. We aimed to determine whether probiotic use is associated with a reduction in severe NEC in infants born <32 weeks gestation. We examine the impact of probiotic exposure on other definitions of NEC, as well as important safety outcomes such as late onset sepsis and mortality. These additional outcomes ensure that any impact on NEC is not being counterbalanced with increased sepsis or mortality.^10^

## Methods

### Study design and data source

We undertook a population retrospective cohort study using existing routinely collected data held in the National Neonatal Research Database (NNRD) and based on a directed acyclic graph (DAG) (Appendix p16, adapted from a previously published DAG^11^). The NNRD contains records for all infants admitted to neonatal units in England and Wales since 2012.^12^ The study design was registered at Clinicaltrials.gov IRAS323099.^13^

### Study population

We extracted data for infants born 23^+0^ to 31^+6^ weeks gestation between January 1st 2016 and December 31st 2022 (seven years) admitted to neonatal units in England and Wales. We excluded infants with the following: missing data for gestational age at birth, birth weight or birth year; missing first admission to neonatal care following birth or missing all data for the first two days of life; birth weight for gestational age absolute z-score exceeds four; died within the first four days of life; major congenital abnormality (Appendix p2).

### Exposures and outcomes

Exposure to probiotics was defined as documented receipt on at least one occasion, of any probiotic in the first 14 days of life. The five products used are listed in Appendix p20. Unexposed to probiotics (comparator) was defined as no documented receipt of probiotics in the first 14 days of life.

The primary outcome was severe NEC, defined as NEC confirmed by laparotomy, histology, or autopsy, or, if no tissue evidence was available, the reported primary cause of death on the death certificate^2^ (NNRD extraction code Appendix p4). Secondary outcomes were.

• Other NEC definitions
  ○ UK National Neonatal Audit Programme (NNAP) defined NEC^14^: NEC diagnosed at postmortem or during surgery or using clinical and radiographic features.
  ○ Pragmatic NEC definition: recorded diagnosis of NEC and received at least 5 consecutive days of antibiotics whilst nil by mouth.
• Late onset sepsis (LOS) primarily defined as a positive blood culture (taken after 72 h of age) of any organism from the NNAP list of “Clearly pathogenic organisms” OR “other organisms” including coagulase negative Staphylococcus (CoNS)^14^ OR a discharge diagnosis (Appendix p8) indicating a “clearly” or “other” pathogenic organism from the NNAP lists.
• Survival to final discharge from neonatal care

Exploratory outcomes included: Severe brain injury, treated retinopathy of prematurity (ROP), bronchopulmonary dysplasia (BPD), severe BPD, time to full feeds, survival without severe NEC, survival without severe NEC or late onset sepsis, survival without NNAP defined NEC (Appendix p10).

### Statistical analysis

The estimated reduction in risk of NEC attributed to probiotics in the most recent Cochrane analysis was 0.54,^3^ and we hypothesised a similar reduction in severe NEC. The UK incidence of severe NEC in this population was 3.2%^2^; we therefore expected the risk in the exposed group to be 1.7%. Assuming two-sided alpha of 0.5%, matching 2500 each of exposed and non-exposed infants would detect this difference with 90% power.

We describe the management of missing data in Appendix p10. The study used propensity matching to control for confounding. We first estimated the probability or ‘propensity’ that each infant would be exposed to probiotics and then matched exposed infants to unexposed infants with similar propensity scores. The DAG (Appendix p16) represents variables that influence probiotic exposure and risk of severe NEC. We assigned a level of importance (*critically, highly or moderately* important) to each variable and determined their availability in the NNRD (Appendix p21).

*Critically important* variables: gestational age (coded in four categories) and birth year (coded in two categories) and *highly important* variables (gestation in days, year of birth, birthweight, level of care on postnatal day three, sex and the neonatal unit's ODN) were automatically included in the propensity score. *Moderately important* variables were selected for inclusion in the propensity score using the step-wise method described by Imbens and Rubin^15^ (Appendix p11). Moderately important variables included: mother's ethnicity, index of multiple deprivation (IMD) quintile, maternal gravidity, multiple birth, maternal infection, antenatal steroids, intrauterine growth restriction (birthweight-for-age z-score <−2^16^), surfactant on day one, any enteral feeds days 1–4 and illness severity. The maximum illness severity score for the first two days of life was scored 0–3, representing the sum of receipt of inotropes, invasive respiratory support or nitric oxide (score one for each).^9^

Matched pairs (exposed versus unexposed to probiotics) with similar propensity scores were created within two *critically important* strata: gestational age (22 + 0 to 24 + 6 weeks, 25 + 0 to 27 + 6 weeks, 28 + 0 to 29 + 6 weeks, 30 + 0 to 31 + 6 weeks) and birth year (2016–2019 or 2020–2022), producing eight groups. We used nearest neighbour (greedy) matching without replacement. Infants were matched in descending order from the highest propensity score. We used the propensity score difference to determine matches and a caliper of 0.2 standard deviations. Our target estimand was the average treatment effect in the treated.

The impact of probiotic exposure was assessed by comparing outcomes between the matched groups using logistic regression. Robust standard errors were used to for correlation within a neonatal unit and within infants born to the same mother. Mortality in the first 28 days after birth was compared using a log rank test and visualised using Kaplan Meier curves. Bonferroni corrections were applied to the analyses of exploratory outcomes.

Systematic reviews suggest the impact of probiotics may differ by degree of prematurity (2). We therefore compared the following outcomes between infants born <28 versus  ≥ 28 weeks gestation: severe NEC, NNAP defined NEC, pragmatically defined NEC, LOS and survival to discharge. In addition, we compared severe NEC between infants with birthweight <1000*g* versus ≥1000*g*.

We conducted three sensitivity analyses. The first sensitivity analysis assessed the impact of the order of data on the matches created by repeating the matching process twenty times, randomising the order of matching. In our second sensitivity analysis, we conducted a logistic regression analysis on the whole cohort without propensity matching, to evaluate whether the findings differed. We adjusted for all the variables included in the propensity score. Our third sensitivity analysis was designed to take account of potential cross-contamination of probiotics between infants cared for in the same neonatal unit. We repeated our main analysis assuming that all infants cared for in a “*probiotic unit”* were exposed to probiotics. A *probiotic unit* was defined as one where:

1. Over 50% of cohort infants born in the same month, who spent day three of life on the unit, were exposed to probiotics in first 14 postnatal days OR
2. At the time the infant was born, the unit had implemented a guideline for routine use of probiotics. Information about unit guidelines was ascertained using a survey.^9^

We restricted this analysis to tertiary Neonatal Intensive Care Units (NICUs) because the smaller number of infants born <32 weeks gestation in non-tertiary units may result in misclassification of a unit's probiotic status.

Two probiotic formulations (LB and PP) were used most commonly. In the subset of infants born during the time period when both probiotic formulations were available (June 2020 to December 2022), we explored the association between probiotic products (LB or PP) and odds of severe NEC. We used multivariable logistic regression and adjusted the analysis for all variables in the propensity score. Robust standard errors were used.

### Additional descriptive results

We report rates of exposure to bovine milk and rates of late onset sepsis using two additional definitions.^14^ We also report rates of severe NEC by sex and by maternal ethnicity (Appendix p9).

### Ethics

Ethics approval was granted by the South-East Scotland Research Ethics Committee 01 (REC reference 23/SS/0016) as part of a larger study evaluating the impact of a care bundle on NEC incidence.^17^

### Patient and public involvement

Focus groups of parents and former NICU patients supported the use of routine data to evaluate the safety and efficacy of probiotics.^18^^,^^19^ The NNRD board, which approved the data extraction, includes two parents of former NICU patients.

### Role of the funding source

The funder of the study had no role in study design, data collection, data analysis, data interpretation, or writing of the report.

## Results

**Fig. 1:**
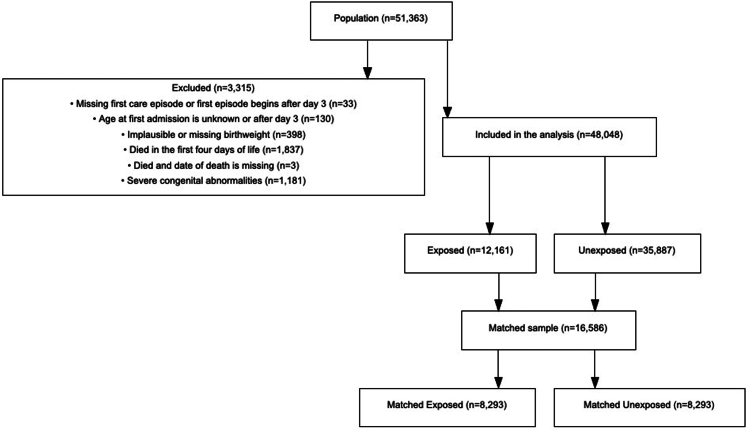
Selection of the propensity matched groups.

Following exclusions (Fig. 1), 48,048 of 51,363 infants born <32 weeks gestation were included in the analyses. 25.3% percent (12,161/48,048) of the cohort infants were exposed and 74.7% (35,887/48,048) unexposed to probiotics in the first 14 days. 16,586 infants (8293 exposed and 8293 unexposed) were included in the matched sample (Fig. 1). Background characteristics of the entire cohort and the matched sample are summarised in Table 1. Background characteristics of infants with complete covariates and incomplete covariates are reported in Appendix p10. The number of infants from each unit is shown in Appendix p22. Propensity score matching resulted in well matched groups (Appendix p18). The median gestational age was 29.4 weeks (IQR 27.4–30.9) for both the matched sample and the whole cohort. Propensity matching occurred across the full range of propensity scores (Appendix p18) and therefore the results should be generalisable to all probiotic exposed infants.

**Table 1:** Key background characteristics of the study participants.

n (%) unless specified	Entire cohort (n = 48,048)			Matched sample (n = 16,586)		
	Exposed (n = 12,161)	Unexposed (n = 35,887)	P value^b^	Exposed (n = 8293)	Unexposed (n = 8293)	P value^b^
*Infant characteristics*						
Gestational age (weeks) Median (IQR)	29.3 (27.3–30.7)	29.6 (27.4–31.0)	<0.00001	29.4 (27.4–30.9)	29.4 (27.4–30.9)	0.65
Sex						
Female	5476 (45.0)	16,219 (45.2)	0.84	3731 (45.0)	3748 (45.2)	0.82
Male	6685 (55.0)	19,668 (54.8)		4562 (55.0)	4545 (54.8)	
Birthweight (g) Median (IQR)	1170 (900–1460)	1220 (919–1510)	<0.00001	1200 (914–1480)	1200 (915–1480)	0.90
Birth year						
2016–2019	4756 (39.1)	24,024 (66.9)	<0.00001	3783 (45.6)	3783 (45.6)	1.00
2020–2022	7405 (60.9)	11,863 (33.1)		4510 (54.4)	4510 (54.4)	
Intrauterine growth restriction	646 (5.3)	1660 (4.6)	0.0024	426 (5.1)	426 (5.1)	1.00
*Infant early care*						
Surfactant on day one after birth						
Received surfactant	6207 (51.0)	19,669 (54.8)	<0.00001	4139 (49.9)	4140 (49.9)	1.00
No surfactant	5952 (48.9)	16,210 (45.2)		4154 (50.1)	4153 (50.1)	
Missing	<5	8		0	0	
Illness severity^a^ (worst of day 1 or 2)						
0	5595 (46.0)	14,298 (39.8)	<0.00001	3884 (46.8)	3903 (47.1)	0.73
1	5118 (42.1)	16,068 (44.8)		3379 (40.7)	3321 (40.0)	
2	1160 (9.5)	4221 (11.8)		849 (10.2)	882 (10.6)	
3	250 (2.1)	863 (2.4)		181 (2.2)	187 (2.3)	
Missing	38 (0.3)	437 (1.2)		0	0	
Highest level of care days 1–4						
Intensive care	10,507 (86.4)	29,665 (82.7)	<0.00001	6961 (83.9)	6949 (83.8)	0.85
High dependency	1463 (12.0)	4997 (13.9)		1165 (14.0)	1184 (14.3)	
Special care/Normal care	190 (1.6)	1224 (3.4)		167 (2.0)	160 (1.9)	
Missing	<5	<5		0	0	
Fed enterally days 1–4	11,481 (94.4)	33,396 (93.1)	<0.00001	7831 (94.4%)	7838 (94.5%)	0.84
*Maternal characteristics*						
Mother's ethnicity						
White	7147 (58.7)	19,990 (55.7)	<0.00001	4989 (60.2)	5050 (60.9)	0.85
Mixed/Multiple ethnic groups	174 (1.4)	645 (1.8)		136 (1.6)	134 (1.6)	
Asian/Asian British	1315 (10.8)	4028 (11.2)		963 (11.6)	924 (11.1)	
Black African/Black Caribbean/Black British	584 (4.8)	2890 (8.1)		430 (5.2)	420 (5.1)	
Other ethnic group	190 (1.6)	792 (2.2)		144 (1.7)	130 (1.6)	
Missing	2757 (22.7)	7542 (21.0)		1631 (19.7)	1635 (19.7)	
IMD Quintile						
1 (Most deprived)	3556 (29.2)	10,599 (29.5)	<0.00001	2460 (29.7)	2424 (29.2)	0.88
2	2332 (19.2)	8019 (22.3)		1715 (20.7)	1728 (20.8)	
3	2138 (17.6)	6078 (16.9)		1576 (19.0)	1624 (19.6)	
4	1879 (15.5)	4876 (13.6)		1417 (17.1)	1404 (16.9)	
5 (Least deprived)	1499 (12.3)	3852 (10.7)		1125 (13.6)	1113 (13.4)	
Missing	757 (6.2)	2463 (6.9)		0	0	
*Pregnancy and birth characteristics*						
Gravidity						
Primigravida	4237 (34.8)	12,278 (34.2)	0.23	2882 (34.8)	2907 (35.1)	0.97
Multigravida	7924 (65.2)	23,609 (65.8)		5411 (65.2)	5386 (64.9)	
Multiple birth						
Singleton	8999 (74.0)	27,073 (75.4)	0.0015	6165 (74.3)	6106 (73.6)	0.30
Multiple	3162 (26.0)	8813 (24.6)		2128 (25.7)	2187 (26.4)	
Missing	0	<5		0	0	
Chorioamnionitis	1899 (15.6)	5383 (15.0)	0.10	1317 (15.9)	1253 (15.1)	0.18
Antenatal steroids						
Any	11,109 (91.3)	32,807 (91.4)	0.60	7612 (91.8)	7619 (91.9)	0.86
None	1022 (8.4)	2957 (8.2)		681 (8.2)	674 (8.1)	
Missing	30 (0.2)	123 (0.4)		0	0	
Perinatal maternal infection	3774 (31.0)	11,770 (32.8)	0.0003	2631 (31.7)	2616 (31.5)	0.82
Neonatal network						
A	477 (3.9)	2635 (7.3)	<0.00001	457 (5.5)	500 (6.0)	0.025
B	1255 (10.3)	2807 (7.8)		1053 (12.7)	1127 (13.6)	
C	1377 (11.3)	2105 (5.9)		1112 (13.)	1117 (13.5)	
D	17 (0.1)	3630 (10.1)		12 (0.1%)	6 (0.1)	
E	351 (2.9)	2108 (5.9)		343 (4.1)	381 (4.6)	
F	533 (4.4)	5861 (16.3)		451 (5.4)	456 (5.5)	
G	1418 (11.7)	940 (2.6)		797 (9.6)	721 (8.7)	
H	144 (1.2)	2971 (8.3)		141 (1.7)	144 (1.7)	
I	1673 (13.8)	1388 (3.9)		929 (11.2)	846 (10.2)	
J	957 (7.9)	3108 (8.7)		851 (10.3)	923 (11.1)	
K	558 (4.6)	1482 (4.1)		9 (0.1)	4 (0.0)	
L	889 (7.3)	4426 (12.3)		738 (8.9)	746 (9.0)	
M	2503 (20.6)	2316 (6.5)		1400 (16.9)	1322 (15.9)	
Missing	9 (0.1)	110 (0.3)		0	0	

a Illness severity scale: 0 = Better condition, 3 = Worse condition. See supplementary materials Appendix 4 for details of how severity is calculated.

b P values are based on Wilcoxon rank-sum test for Gestational age and Birthweight. All other variables use Chi-squared test. Where the number of infants is in the range 1–4, these are reported as “<5”.

In the propensity matched analysis, the incidence of severe NEC, the primary outcome, among infants exposed to probiotics versus unexposed was 3.3% (277/8293) versus 4.2% (345/8293) (OR 0.80, 95% CI 0.72–0.89). Results for secondary outcomes and other exploratory outcomes are presented in Table 2. A Kaplan Meier plot of survival in the first 28 days of life showed strong evidence of differences in the risk of mortality over time, as a function of probiotic exposure, χ^2^ = 77.0, d.f. = 1, p < 0.001 (Appendix p19).

**Table 2:** Neonatal outcomes in the matched sample (n = 16,586) and by gestational age group.

	All gestational ages			Gestational age <28 weeks			Gestational age ≥28 weeks		
	Exposed (n = 8293)	Unexposed (n = 8293)	Odds ratio (95% CI)	Exposed (n = 2513)	Unexposed (n = 2513)	Odds ratio (95% CI)	Exposed (n = 5780)	Unexposed (n = 5780)	Odds ratio (95% CI)
	n (%)	n (%)		n (%)	n (%)		n (%)	n (%)	
Primary outcome									
Severe NEC^a^	277 (3.3)	345 (4.2)	0.80 (0.72–0.89)	219 (8.7)	247 (9.8)	0.88 (0.82–0.93)	58 (1.0)	98 (1.7)	0.59 (0.47–0.73)
Secondary outcomes									
NNAP defined NEC^b^	360 (4.6)	428 (5.6)	0.82 (0.75–0.90)^c^	248 (10.4)	268 (11.4)	0.90 (0.85–0.96)^c^	112 (2.1)	160 (3.0)	0.69 (0.60–0.79)^c^
Pragmatically defined NEC^b^	518 (6.3)	643 (7.8)	0.79 (0.70–0.89)^c^	367 (14.9)	401 (16.2)	0.90 (0.83–0.99)	151 (2.6)	242 (4.2)	0.61 (0.53–0.71)^c^
Late onset sepsis	896 (10.8)	951 (11.5)	0.94 (0.90–0.97)^c^	615 (24.5)	636 (25.3)	0.96 (0.94–0.97)^c^	281 (4.9)	315 (5.4)	0.89 (0.81–0.97)^c^
Survival to discharge	8013 (96.6)	7813 (94.2)	1.76 (1.65–1.88)^c^	2288 (91.0)	2122 (84.4)	1.87 (1.74–2.02)^c^	5725 (99.0)	5691 (98.5)	1.63 (1.41–1.89)^c^
Exploratory outcomes									
Survival without severe NEC	7846 (94.6)	7602 (91.7)	1.60 (1.48–1.72)^d^	2159 (85.9)	1979 (78.8)		5687 (98.4)	5623 (97.3)	
Survival without severe NEC or late onset sepsis	7591 (91.5)	7344 (88.6)	1.40 (1.31–1.50)^d^	1978 (78.7)	1807 (71.9)		5613 (97.1)	5537 (95.8)	
Survival without NNAP NEC^b^	7278 (93.0)	6943 (89.4)	1.56 (1.46–1.66)^d^	1997 (83.3)	1803 (75.1)		5281 (97.2)	5140 (95.7)	
Severe brain injury	575 (6.9)	711 (8.6)	0.79 (0.75–0.85)^d^	379 (15.1)	478 (19.0)		196 (3.4)	233 (4.0)	
Treated retinopathy of prematurity^b^^,^^e^ [live n]	345 (4.3) [8101]	309 (3.9) [7907]	1.09 (1.03–1.17)^d^	314 (13.4) [2347]	279 (12.8) [2188]		31 (0.5) [5754]	30 (0.5) [5719]	
Bronchopulmonary dysplasia^b^^,^^e^ [live n]	2622 (32.8%) [7991]	2396 (30.7%) [7818]	1.11 (1.06–1.15)^d^	1564 (68.1) [2296]	1449 (67.7) [2140]		1058 (18.6) [5695]	947 (16.7) [5678]	
Severe bronchopulmonary dysplasia^b^^,^^e^ [live n]	119 (1.5) [7991]	166 (2.1) [7818]	0.70 (0.66–0.74)^d^	85 (3.7) [2296]	123 (5.8) [2140]		34 (0.6) [5695]	43 (0.8) [5678]	
Time to full feeds (days) Median (IQR)	12.0 (10.0–16.0)	13.0 (10.0–18.0)	0.90 (0.87–0.92)^d^	16 (13–22)	19 (14–28)		12 (9–14)	12 (9–15)	

a Analyses of the primary outcome are not adjusted for multiple testing.

b Rates of missing data in the matched sample: NNAP defined NEC = 6.4%, Pragmatically defined NEC = 1.2%, Survival without any NEC = 6.0%, Treated ROP = 3.5%, Bronchopulmonary dysplasia = 4.7%, Severe bronchopulmonary dysplasia = 4.7%, Time to full feeds = 4.4%. Percentages in the table calculated using number with non-missing data as the denominator.

c Significant at the 5% level after correction for multiple testing i.e. uncorrected p-value was less than 1.25% (5%/4).

d Significant at the 5% level after correcting for multiple testing i.e. uncorrected p-value was less than 0.63% (5%/8).

e Because these outcomes are assessed after birth, the denominators for percentages reflect the number of infants alive at the corrected gestational age when the outcome would normally be assessed. For retinopathy of prematurity first assessment would be at the later of 31 weeks corrected gestational age or 4 weeks after birth. For bronchopulmonary dysplasia assessment would be at 36 weeks corrected gestational age. The numbers of infants alive at the anticipated assessment date, with non-missing outcome data, are shown in square brackets.

**Fig. 2:**
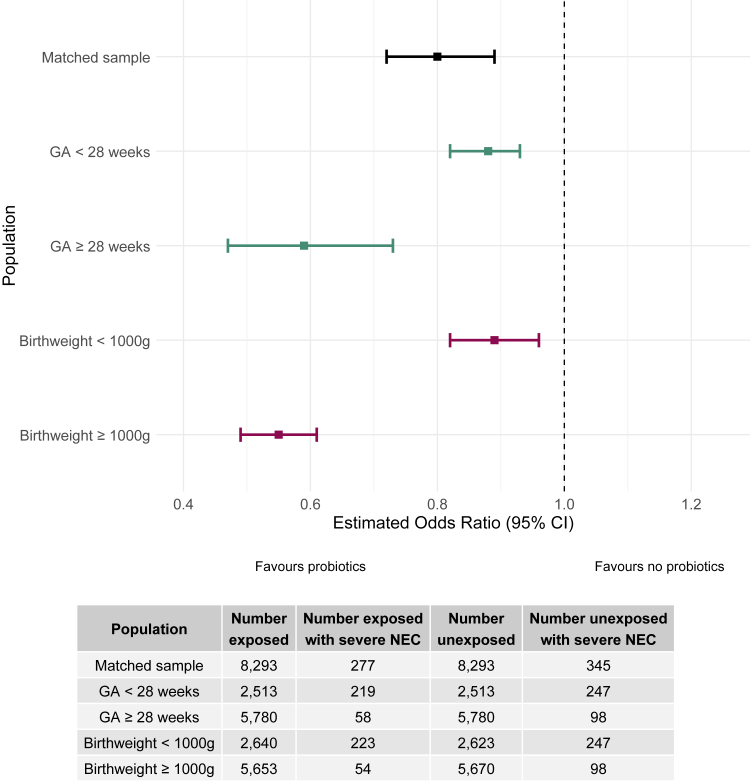
Forest plot presenting the summary of odds ratios and 95% confidence intervals for the association between probiotics and severe necrotising enterocolitis within the matched sample.

In the subgroup analyses of the two gestational age groups, NEC, using all three definitions, remained lower in both groups of infants exposed to probiotics (Table 2). Severe NEC among infants <28 weeks gestation was 8.7% (219/2513) in the exposed group versus 9.8% (247/2513) in the unexposed (OR = 0.88, 95% CI 0.82–0.93). In the subgroup ≥28 weeks gestation, the incidence of severe NEC was 1.0% (58/5780) in exposed infants versus 1.7% (98/5780) in the unexposed (OR = 0.59, 95% CI 0.47–0.73). In the birthweight subgroup analyses, among infants born weighing <1000*g*, incidence of severe NEC was 8.4% (223/2640) for exposed infants versus 9.4% (247/2623) for unexposed infants (OR = 0.89, 95% CI 0.82–0.96). Among infants with a birthweight ≥1000*g* incidence of severe NEC was 1.0% (54/5653) for exposed infants and 1.7% (98/5670) in the unexposed group (OR = 0.55, 95% CI 0.49–0.61) (Fig. 2).

In the sensitivity analysis that examined the impact of match ordering, results were similar to the primary analysis. The median odds ratio for the effect of probiotic exposure on severe NEC was 0.78 (IQR 0.77–0.79). An analysis of the whole cohort (12,161 exposed and 35,887 unexposed infants) without propensity matching yielded similar findings to the main analysis, but with wider confidence intervals for severe NEC (OR 0.82, 95% CI 0.67–0.99) and all secondary outcomes (with the exception of late onset sepsis where there was no evidence of an association with probiotic exposure) (Appendix p24). Our final sensitivity analysis, where exposure was assigned on the basis of neonatal unit included 7596 infants from 37 tertiary NICUs; 3798 in probiotic units and 3798 in non-probiotic units. Infants cared for in a probiotic NICU had 5% (189/3798) severe NEC compared to 5.7% (215/3798) of infants treated in a non-probiotic NICU (OR = 0.87, 95% CI 0.86–0.89).

The pattern of use of different probiotic products over time has been previously reported.^9^ Of 12,161 infants in the whole cohort who were exposed to probiotics, 8036 (66.1%) were given just LB. PP began to be used in the UK in June 2020 and was given as the sole probiotic to 983 infants (8.1%) of the whole cohort. Analysing infants born from June 2020 and exposed to LB or PP only (n = 5398, exposed to LB = 4415 versus exposed to PP = 983), incidence of severe NEC in infants exposed to LB was 2.7% (95% CI: 2.2%–3.2%) versus 5.1% in infants exposed to PP (95% CI: 3.7%–6.5%) (adjusted OR = 0.89, 95% CI 0.68–1.16) suggesting no significant difference between formulations in their impact on severe NEC.

## Discussion

For most clinicians, the key drivers for administration of probiotics to preterm infants are to reduce NEC, late onset sepsis and mortality. We designed this large population study to examine the primary outcome of severe NEC (defined as NEC confirmed by laparotomy, histology, or autopsy, or, if no tissue evidence was available, the reported primary cause of death on the death certificate). Using a propensity-matching approach, we found that probiotic exposure was associated with a reduction in NEC, consistent across all three definitions, including severe NEC, NEC defined by clinical and radiological features, and pragmatic NEC. Our findings were similar for infants born < and ≥28 weeks gestation, when we applied logistic regression without propensity matching to the entire cohort, and when assignment of probiotic exposure was at unit rather than baby level.

We also found an association between probiotics and a reduction in LOS and mortality. However, results for secondary and exploratory outcomes, even though corrected for multiple testing, should be interpreted with caution. Of note, the effect difference in mortality was seen early (Figure S4). In the full cohort, the median day of starting probiotics was day 5 (IQR 2–8)^9^ and whilst it is possible that probiotics may exert a beneficial effect within a short number of days exposure^20^ the currently understood mechanisms of action make very rapid onset of benefit improbable. We therefore believe it is unlikely that probiotics are directly responsible for the very early reduction in mortality observed in this study.

Our findings align in part with the Cochrane meta-analysis^3^; however we found evidence of a small but clinically important benefit for extremely preterm and VLBW infants, which Cochrane did not. The Cochrane authors acknowledge the low certainty of evidence due to limited numbers of separately reported, extremely preterm infants (n = 1836) within included RCTs. Since the Cochrane review, a number of network and umbrella meta-analyses have concluded that probiotics decrease the risk of NEC and mortality.^21^, ^22^, ^23^ A recent large Canadian retrospective observational study found an association between probiotics and reduced mortality, but no reduction in rates of NEC or late-onset sepsis, including among the subgroup born <1000*g*.^24^ Whilst sample sizes in the Canadian study were similar to ours, methodological differences, including the population inclusion criteria, NEC definition and propensity score variables, may explain the wider confidence intervals and non-significant results. The question about efficacy in more immature infants is important and may be addressed in the ongoing Probiotics in Extreme Prematurity (PEPS) trial (NCT05604846).

In this study, we explored the effect of the two most commonly used probiotics in the UK, only one of which meets current ESPGHAN recommendations (PP).^6^ Based on the event rates associated with the analysis of PP versus LB, a future randomised controlled trial comparing these two products would need to recruit 1000 infants per arm to detect an absolute risk difference of 2.4% (from 5.1% to 2.7%) in severe NEC with 80% power. Prior to designing future head to head trials of probiotic products, research is necessary to understand the shared mechanisms of action of probiotics.^25^ In this study, we have demonstrated the utility of real-world, large datasets to add to the literature on this important topic. This may be particularly relevant in addressing the uncertainties of probiotic use in preterm infants, as relying on the gold-standard RCT methodology to compare the many potential probiotic strains and combinations, may be practically and economically challenging.^6^

The strengths of this study include an *a priori* study design.^13^ We excluded infants who died in the first four days because probiotic exposure may be influenced by perceived chances of survival^9^ and early deaths are unlikely to be related to a brief exposure to probiotics. We used a DAG to inform the selection of variables eligible for inclusion in the propensity score. Variables available in the NNRD enabled us to apply a comprehensive propensity matching approach,^15^ resulting in well matched groups, whilst maintaining sample sizes that are larger than all single RCTs and the Cochrane meta-analysis.^3^ Restricting the exposure period to the first 14 days avoids the situation where the exposure is influenced by the outcome, since NEC typically occurs after the first two weeks of life.^26^ The use of severe NEC, with its robust definition, as the primary outcome, reduces the likelihood of outcome misclassification. However, we acknowledge the possibility that some deaths deemed to be caused by NEC, but not confirmed by laparotomy or postmortem, may be misclassified. We also have confidence that our measure of probiotic exposure is subject to minimal misclassification since, for most infants, exposure was sustained. Probiotic mechanisms, especially those that may be specific to a select strain as opposed to more generic probiotic properties, are poorly understood,^25^ but this study included receipt of any available product, and shows overall benefit.

We highlight the following as limitations of this study. Whilst restricting the exposure period to the first 14 days was intended to minimise the chances of the outcome e.g. NEC occurring before the exposure, we cannot exclude this possibility. Defining the exposure period as the first 14 days may also introduce bias in the analysis of mortality as the pre-treatment period is immortal for the treated group. We attempted to mitigate this with our unit level sensitivity analysis and by excluding infants who died in the first four days of life; however this limits the generalisability of our study findings to infants who survive the very early postnatal period.

Despite the use of the DAG within this observational study, it remains possible that there are unobserved confounding factors or residual confounding and furthermore, there may remain selection bias in the 25% of babies exposed to probiotics versus those not exposed. We did not match by unit, as babies who do and do not receive probiotics in a probiotic using unit have been shown to be different.^9^ We did match by geographically defined networks, to control for clinical practices that are likely to be informed by network guidelines and are not otherwise accounted for by the DAG. This includes choice of feed type, including donor milk use. Feed type is a complex daily variable involving both different feed types, fortifiers and volumes (not consistently recorded in NNRD) that could not be meaningfully incorporated into our analysis. The NNRD does not include data on infant ethnicity and available data on maternal ethnicity has substantial levels of missingness. We chose not to impute maternal ethnicity, as studies of ethnicity recording in other UK health databases suggest missingness is not random.^27^ Other variables had substantially lower levels of missingness, however these may still be a source of bias.

The illness severity score used here, whilst associated with mortality in this cohort,^9^ has not otherwise been validated. The NNRD does not consistently identify the timing of diagnoses or procedures. This means that factors which may influence probiotic receipt, for example early surgery or sepsis, cannot be accounted for. Despite our attempts to capture LOS using a range of NNRD variables, LOS may still be under-reported; furthermore our definition of LOS does not allow reporting of the prevalence of specific infecting organisms, including of administered probiotic bacteria. Data on the NNRD are extracted from electronic patient records used for clinical care rather than primary research purposes. There is limited real-time data validation and we acknowledge potential for data inaccuracies and misdiagnosis in outcomes, particularly NEC versus spontaneous intestinal perforation, given the diagnosis can be challenging even at surgery. Furthermore, data entry generally does not require confirmation that relevant conditions have not occurred, meaning that some cases of important outcomes in this study may have been missed.

The pros and cons of regulation of probiotics as a medicinal product by bodies such as the FDA in the US continues to be a much debated topic. ESPGHAN and the European Foundation for the Care of Newborn Infants (EFCNI) published a statement observing that ‘abandoning available evidence based products while waiting for pharmaceutical grade probiotic products would cost many lives’ and ‘restricting to future FDA approved products would likely significantly increase costs’.^28^ Verification of the presence and concentration of an intended strain may not reduce the risk of probiotic sepsis, which may be from gut translocation or line contamination and is unrelated to the ‘quality’ of the product.

Given the heterogeneity in probiotic strains, regulatory processes and probiotic availability unique to each country, clinicians should monitor and evaluate the safety and efficacy of probiotics used in their own neonatal units. Our findings from this population-level analysis help inform discussions between clinicians and families when weighing up the benefits and risks associated with the use of probiotics.

### Conclusion

This very large study demonstrates important survival and morbidity benefits for preterm infants <28 weeks and <32 weeks gestation, who receive probiotics or are cared for in a centre that uses them. These data are subject to all the limitations of observational studies. However, the findings are consistent with the Cochrane meta-analysis, and we did not find evidence of harm (NEC, late onset sepsis and mortality). We recommend that neonatal units examine their own rates of NEC, LOS and mortality and some may wish to consider implementing probiotics whilst awaiting further data. Probiotics used should meet appropriate professional recommendations and/or regulatory requirements where these exist, and information shared with parents.

## Contributors

CB, SO, LS and PF were involved in the study inception and submitted for research regulatory approvals. AA, CB, and LS planned statistical analysis, and together with SO, had access to the raw data. AA, CB, JB, SO, LS, KC and PF all contributed to overall study design, protocol development and the writing and review of this paper. All authors have approved the final version for submission. CB is the guarantor.

## Data sharing statement

Pseudonymised data used in this study are held by the National Neonatal Research Database and may be requested via the Health Data Research UK digital gateway. Data will be available after approval of the proposal and execution of a data access agreement. The data dictionary for this study is included in Appendix p21.

## Declaration of interests

CB is supported through a UK National Institute for Health and Care Research (NIHR) Advanced Fellowship personal award (NIHR300617) and has current research grants from the NIHR and Medical Research Council (MRC). CB is a member of the NIHR Team Science selection panel committee. CB has received honoraria as a PhD examiner from Cork University, Chinese University, University College London, University of Helsinki in the last three years. CB is a member of the Data monitoring committee for clinical trial “PAPAGAIO - Diagnosis: Placental Growth Factor testing to diagnose preeclampsia in low- and middle-income countries. CB is British Association of Perinatal Medicine Honorary Secretary and a member of the NHS England Research Advisory Board. KC is Chair of the Data Safety Monitoring Board (DSMB) for the RESPONSE trial (Medical Research Council funded) and member of the Data Monitoring and Ethics committee (DMEC) for the Big Baby Obstetric trial, NIHR funded. AA is funded by an Imperial College PhD studentship and has previously received Wells Fargo stock as part of prior employment. PF has have received honoraria as a PhD examiner from Imperial College London, Newcastle University and the Royal College of Surgeons of Ireland in the last five years. PF is a member of the Data Monitoring Committee for clinical trial MOLI: an obstetric trial evaluating at home induction of labor using mifepristone, funded by Nordic Pharma. PF is a Trial Steering Committee Member for studies SURFOn (NIHR funded RCT) and RESPONSE (Medical Research Council funded). JB has a patent registered through Newcastle university ref number PCT/GB2024/050842 June 2024 for a possible probiotic. JB is previous member of Data Monitoring Board for African probiotic study. JB is medical advisor to NEC UK charity and research lead for N3 (Neonatal nutrition network). SO has current research grants from the NIHR and the MRC.
