# Supplementary material for: Evaluating the effect of probiotics on severe necrotising enterocolitis in preterm infants born before 32 weeks gestation in England and Wales: a propensity-matched population study
Source: Lancet Reg Health Eur. 2025 Dec 22;62:101571. doi: 10.1016/j.lanepe.2025.101571 (PMC12796542; doi:10.1016/j.lanepe.2025.101571)
Supplement: Supplementary Figures and Tables [file mmc1.docx]

**SUPPLEMENTARY APPENDIX**

| **Supplementary materials** | | **Page number** |
| --- | --- | --- |
| 1 | Major congenital abnormalities | 2 |
| 2 | Definition of NEC using NNRD variables | 4 |
| 3 | Additional descriptive results | 8 |
| 4 | NEC in subgroups of the whole cohort defined by sex, maternal ethnicity and intra-uterine growth restriction. | 9 |
| 5 | Definitions of exploratory outcomes | 10 |
| 6 | Treatment of missing data | 10 |
| 7 | Building the propensity score model | 11 |
| 8 | Acknowledgements | 12 |
| **Supplementary figures** | |  |
| Figure S1 | Directed acyclic graph | 16 |
| Figure S2 | Balance plot showing standardised differences between probiotic groups before and after propensity matching | 17 |
| Figure S3 | Distribution of propensity scores | 18 |
| Figure S4 | Kaplan meier plot of survival by probiotic exposure | 19 |
| **Supplementary tables** | |  |
| Table S1 | Probiotics products used by neonatal units during the study period | 20 |
| Table S2 | Concepts from the DAG and the NNRD variables used to operationalise those concepts | 21 |
| Table S3 | Number of babies contributed by each neonatal unit | 22 |
| Table S4 | Results of the analysis of the full cohort | 24 |

1. **MAJOR CONGENITAL ABNORMALITIES**

**Major congenital gastrointestinal malformations**

Correction of congenital atresia of oesophagus, oesophageal atresia, oesophageal atresia with distal tracheo-oesophageal fistula, oesophageal atresia with tracheoesophageal fistula, oesophageal atresia without distal fistula, oesophageal atresia without tracheoesophageal fistula, thoracotomy and repair of oesophageal atresia and tracheo-oesophageal fistula with primary anastomosis, atresia and stenosis of small intestine, atresia and stenosis of duodenum, duodenal atresia / stenosis / web (specify), duodenal atresia / stenosis / web, duodenal atresia / stenosis, duodenal atresia, atresia and stenosis of ileum, ileal atresia / stenosis (specify), ileal atresia / stenosis, jejunal atresia / stenosis (specify), jejunal atresia / stenosis, atresia and stenosis of large intestine, congenital absence atresia / stenosis parts of large intestine, congenital absence atresia / stenosis parts of large intestine, congenital absence atresia / stenosis of rectum with fistula, congenital absence atresia / stenosis rectum without fistula, congenital absence atresia / stenosis anus with fistula, congenital absence atresia / stenosis anus without fistula, congenital absence atresia / stenosis of large intestine part unspecified, atresia of oesophagus without fistula, atresia of oesophagus with tracheo-oesophageal fistula (tof), recurrent tracheo-oesophageal fistula, tracheo-oesophageal fistula (h-type), congenital tracheo-oesophageal fistula without atresia (tof), congenital stenosis and stricture of oesophagus, congenital stenosis of the oesophagus, congenital oesophageal web, oesophageal web, large bowel or rectum - atresia, high anorectal anomaly with rectourethral fistula, high anorectal anomaly with rectovesical fistula, high anorectal anomaly with rectocutaneous fistula, high anorectal anomaly with rectocloacal fistula, high anorectal anomaly with fistula (specify), high anorectal anomaly without fistula, anorectal anomaly - high without fistula, low anorectal anomaly with anocutaneous fistula, low anorectal anomaly with anovestibular fistula, low anorectal anomaly with fistula (other specify), congenital absence atresia / stenosis anus without fistula, anus - imperforate, imperforate anus, low anorectal anomaly without fistula, low anorectal anoma, congenital anal stenosis, persistent cloaca, exomphalos (major), exomphalos (minor), exomphalos malrotation, exomphalos, omphalocele, closure of gastroschisis includes closure of exomphalos, primary repair exomphalos, repair exomphalos using prosthesis (specify type), gastroschisis, delayed closure gastroschisis, primary repair gastroschisis, repair gastroschisis using prosthesis (specify type), silo insertion for reduction of gastroschisis, delayed closure exomphalos, cutback of covered anus, repair of imperforate anus (with or without vaginal, cutback of low anorectal anomaly (nixon), oesophageal atresia - repair of anastomotic leak, primary repair of oesophageal atresia, closure of recurrent tracheo-oesophageal fistula, closure of tracheooesophageal fistula, closure of tracheo-oesophageal fistula, duodenal atresia/stenosis repair, duodenal atresia/stenosis repair (von)

**Other severe congenital conditions, lethal or requiring early surgical intervention**

*Cardiac and circulatory system*

Congenital malformations of cardiac chambers and connections, common arterial trunk (truncus malformation), truncus arteriosus, double outlet right ventricle (dorv), double outlet left ventricle (dolv), dextrotransposition of aorta, transposition great arteries (tga), transposition of the great vessels (tga), double inlet ventricle (dilv), discordant atrioventricular connection, isomerism of atrial appendages, atrial isomerism & asplenia, atrial isomerism with asplenia, atrial isomerism with polyspenia, atrial isomerism, other congenital malforms of cardiac chambers and connections, congenital malforms of cardiac chambers and connections unspec, complete atrioventricular septal defect, atrio-ventricular septal defect (avsd), atrioventricular septal defect (avsd), tetralogy of fallot, atrium single, ventricle single, congenital malformations of pulmonary and tricuspid valves, pulmonary valve atresia, congenital pulmonary valve stenosis, pulmonary valve stenosis (ps), congenital pulmonary valve insufficiency, other congenital malformations of pulmonary valve, congenital tricuspid atresia / stenosis, ebstein's anomaly, hypoplastic right heart syndrome, other congenital malformations of tricuspid valve, congenital malformation of tricuspid valve (unknown or unspecified cause), congenital malformations of aortic and mitral valves, congenital stenosis of aortic valve (as), bicuspid aortic valve, mitral atresia, congenital insufficiency of aortic valve, congenital mitral stenosis (ms), hypoplastic left heart syndrome (hlh), other congenital malformations of aortic and mitral valves, congenital malformation of aortic and mitral valves unspec, coarctation of aorta, coarctation of the aorta, stenosis of aorta (as), other malformation of aorta, malformation of aorta, double aortic arch, hypoplasia of aortic arch, interrupted aortic arch, atresia of pulmonary artery, pulmonary stenosis (physiological branch stenosis), pulmonary stenosis - branch, other congenital malformations of great arteries, total anomalous pulmonary venous connection (tapvd), total anomylous pulmonary venous drainage (tapvd), blalock-taussig shunt

*Respiratory system, including diaphragmatic hernia*

Choanal atresia - bilateral, choanal atresia - unilateral (l), choanal atresia - unilateral (r), choanal atresia / stenosis (specify), choanal stenosis, congenital malformations of trachea and bronchus, congenital tracheomalacia, tracheomalacia, other congenital malformations of trachea, tracheal agenesis or atresia, bronchomalacia, congenital malformations of bronchus, congenital cystic lung (ccam), congenital cystic lung (congenital lobar emphysema), congenital cystic lung, sequestration of lung, congenital bronchiectasis, hypoplasia and dysplasia of lung, repair choanal atresia, congenital diaphragmatic hernia, congenital diaphragic hernia, morgagni diaphragmatic hernia, diaphragmatic hernia - left, diaphragmatic hernia - right, recurrent congenital diaphragmatic hernia, eventration of diaphragic hernia, eventration of the diaphragm, repair of congenital diaphragmatic hernia, prosthetic repair of congenital diaphragmatic hernia (specify), aplasia of the diaphragm, fetoscopic insertion of tracheal plug for congenital diaphragmatic hernia, other repair of diaphragmatic hernia (specify), other specified repair of diaphragmatic hernia, repair of diaphragmatic hernia using abdominal approach nec, primary repair of congenital diaphragmatic hernia, thoracoscopic repair of congenital diaphragmatic

*Brain and nervous system*

Does not include spina bifida occulta

Frontal encephalocele, nasofrontal encephalocele, occipital encephalocele, encephalocoele - occipital, encephalocele (unknown or unspecified cause), encephalocele, meningocele (specify site), myelomeningocele (specify site), meningocele & hydrocephalus (specify site), thoracic spina bifida with hydrocephalus, lumbar spina bifida with hydrocephalus, sacral spina bifida with hydrocephalus, (unknown or unspecified cause) spina bifida with hydrocephalus, cervical spina bifida without hydrocephalus, thoracic spina bifida without hydrocephalus, lumbar spina bifida without hydrocephalus, sacral spina bifida without hydrocephalus, spina bifida (unknown or unspecified cause), spina bifida, repair of spina bifida, repair of encephalocele, anencephaly and similar malformations, anencephaly, craniorachischisis, iniencephaly, holoprosencephaly, closure of spinal myelomeningocele, closure of spinal meningocele

*Urinary system*

Bilateral renal agenesis, renal agenesis, bilateral, potter's syndrome, autosomal recessive polycystic kidney - infantile, polycystic kidney, infantile type, autosomal dominant polycystic kidney in childhood, polycystic kidney, adult type, polycystic kidney, exstrophy of urinary bladder, bladder exstrophy, posterior urethral valves (puv), congenital posterior urethral valves (puv), congenital absence of bladder and urethra

*Other miscellaneous lethal conditions*

Thanatophoric short stature, Edwards syndrome (trisomy 18), Edwards syndrome (unknown or unspecified cause), trisomy 18, Patau syndrome (trisomy 13), trisomy 13, sirenomelia, triploidy and polyploidy

**2. DEFINITION OF NEC USING NNRD VARIABLES**

**A) Severe NEC**

This method collects data from 3 tables:

- Episode
- Daily summary
- Abdoxray

Multiple fields are used to collect data about NEC diagnosis. We define Severe NEC as true if any of the following conditions (1-8) are met in any of the following tables:

**Within the Episode table:**

1. NEC is listed as ‘Cause of Death’
2. ‘Postmortem confirmation’ is true.
3. Any of the following in ‘Gastrointestinal Diagnoses’, ‘Principle Procedures during stay’ or ‘Principal Diagnosis at discharge’:

- LAPAROTOMY
- LAPAROTOMY APPROACH NEC
- COLECTOMY AND ILEOSTOMY NEC

**AND**

Any of the following in ‘Gastrointestinal Diagnoses’ or ‘Principal Diagnosis at discharge’:

- NECROTISING ENTEROCOLITIS
- NECROTIZING ENTEROCOLITIS
- NECROTIZING ENTEROCOLITIS – CONFIRMED
- NECROTISING ENTEROCOLITIS – CONFIRMED
- NECROTISING ENTEROCOLITIS – PERFORATED
- NECROTIZING ENTEROCOLITIS – PERFORATED
- NECROTISING ENTEROCOLITIS - PROVEN (ON XRAY OR AT SURGERY)
- NECROTIZING ENTEROCOLITIS - PROVEN (ON XRAY OR AT SURGERY

1. Any of the following in ‘Gastrointestinal Diagnoses’ or ‘Principal Diagnosis at discharge’:

- NECROTISING ENTEROCOLITIS
- NECROTIZING ENTEROCOLITIS
- NECROTIZING ENTEROCOLITIS – CONFIRMED
- NECROTISING ENTEROCOLITIS – CONFIRMED
- NECROTISING ENTEROCOLITIS – PERFORATED
- NECROTIZING ENTEROCOLITIS – PERFORATED
- NECROTISING ENTEROCOLITIS - PROVEN (ON XRAY OR AT SURGERY)
- NECROTIZING ENTEROCOLITIS - PROVEN (ON XRAY OR AT SURGERY

**AND**

‘Discharge Destination’ listed as Death.

1. Any of the following in ‘Gastrointestinal Diagnoses’ or ‘Principal Diagnosis at discharge’:

NECROTISING ENTEROCOLITIS – PERFORATED

NECROTIZING ENTEROCOLITIS – PERFORATED

**Within the Daily summary table**

1. Surgery is listed on any day in ‘NEC Treatment’.

**AND**

**Within the Episodes table**

Any of the following in ‘Gastrointestinal Diagnoses’ or ‘Principal Diagnosis at discharge’:

- NECROTISING ENTEROCOLITIS
- NECROTIZING ENTEROCOLITIS
- NECROTIZING ENTEROCOLITIS – CONFIRMED
- NECROTISING ENTEROCOLITIS – CONFIRMED
- NECROTISING ENTEROCOLITIS – PERFORATED
- NECROTIZING ENTEROCOLITIS – PERFORATED
- NECROTISING ENTEROCOLITIS - PROVEN (ON XRAY OR AT SURGERY)
- NECROTIZING ENTEROCOLITIS - PROVEN (ON XRAY OR AT SURGERY

**Within the Abdoxray table**

1. ‘Yes’ is listed under ‘Laparotomy Performed’ and ‘Yes’ listed in ‘Histology Confirmation NEC’
2. ‘Yes’ listed in ‘Visual Inspection Confirmation NEC’

**B) NNAP defined NEC**

The NNAP definition of NEC, first ensures infants are born at less than 32 weeks gestation and survived to 48 hours using data from the Episodes table (Variables admittimeanon and dischtimeanon).

Once the cohort of infants is established, we categorised NEC using the ‘NEC diagnosed at discharge’ fields from the Episodes table.

/*Confirmed NEC*/

*NEC diagnosis confirmed by clinical signs;

if NECDiagnosis = **1** and NECDiagBasedOn =**10** and clinicalfeatures ^= **.** and radiographicfeatures ^ = **.** then NEC=**8**;

if NECDiagnosis = **1** and NECDiagBasedOn =**11** then NEC=**8**;* NEC confirmed by surgery;

if NECDiagnosis = **1** and NECDiagBasedOn =**12** then NEC=**8**; *NEC confirmed by postmortem;

if NECDiagBasedOn=**10** and clinicalfeatures ^= **.** and radiographicfeatures ^ = **.** then NEC=**8**; *clinical signs;

if NECDiagBasedOn in (**11**,**12**) then NEC=**8**; *postmortem or surgery;

/*No NEC*/

if NECDiagnosis =**0** then do;

if finaldischarge = **3** then NEC=**7**; *No NEC but died;

if finaldischarge ^=**3** then NEC=**6**; *No NEC but didnt die;

Where NECdiagnosis is ticked yes but missing clinical/radiographic features confirmation this was treated as No NEC.

Where NEC diagnosis is ticked yes but no further basis for that diagnosis, treated as no NEC

if NECDiagnosis = **1** and NECDiagBasedOn = **.** and finaldischarge= **3** then NEC=**4**; /*Where infant died*/

Infants with no NEC data (NNRD fields NECDiagnosis and NECDiagBasedOn ) entered are treated as 'Missing’

Once an episodic view of the NEC variables has been captured, it is now necessary to look across all the episodes of a infant for a confirmed NEC diagnosis:

Maximise NEC status over all episodes for each infant. Coded as

Missing if NEC_Diagnosis = **0** then Final_NEC=**0**; OR if NEC_Diagnosis = **1** then Final_NEC=**1**;

NO NEC if NEC_Diagnosis in (**2**,**3**,**6**) then Final_NEC=**2**; OR if NEC_Diagnosis in (**4**,**5**,**7**) then Final_NEC=**3**; /*No NEC but died*/

NEC present if NEC_Diagnosis = **8** then Final_NEC=**4**

**C) Pragmatic NEC**

We define pragmatic NEC as present if either of the following conditions are met in any of the following tables:

Within the Daily summary table:

1. Surgery or conservative treatment is listed on any day in ‘NEC Treatment’

**AND**

Being nil by mouth (confirmed by ‘DayEnteralFeeds’, ‘FormulaName’, ‘FeedingMethod’, ‘VolumeMilk’) and any of the following in ‘DrugsDay’ for 5 **consecutive** days:

- 'BENZYL PENICILLIN'
- 'AUGMENTIN'
- 'FLUCLOXICILLIN'
- 'FLUCLOXACILLIN'
- 'GENTAMICIN'
- 'CO-AMOXICLAV'
- 'COAMOXICLAV'
- 'CIPROFLOXACIN'
- 'NETILMICIN'
- 'AMIKACIN'
- 'TAZOCIN'
- 'METRONIDAZOLE'
- 'VANCOMYCIN'
- 'CEFOTAXIME'
- 'AMPICILLIN'
- 'CEFUROXIME'
- 'CEFTAZIDIME'
- 'CEFTRIAXONE'
- 'PIPERACILLIN'
- 'OFLACILLIN'
- 'AZLOCILLIN'
- 'LINEZOLID'
- 'CEFALEXIN'
- 'AMOXICILLIN'
- 'MEROPENEM'
- 'IMEPENEM'
- 'IMIPENEM'

Within the Daily summary table:

1. Any of the following in ‘DiagnosesDay’:

- NECROTISING ENTEROCOLITIS*
- NECROTIZING ENTEROCOLITIS*

**OR**

Any of the following in ‘Code’ in the Diagnosis table:

- **1010683**
- **10708**
- **15809**

**AND**

Being nil by mouth (confirmed by ‘DayEnteralFeeds’, ‘FormulaName’, ‘FeedingMethod’, ‘VolumeMilk’) and any of the following in ‘DrugsDay’ for 5 **consecutive** days:

- 'BENZYL PENICILLIN'
- 'AUGMENTIN'
- 'FLUCLOXICILLIN'
- 'FLUCLOXACILLIN'
- 'GENTAMICIN'
- 'CO-AMOXICLAV'
- 'COAMOXICLAV'
- 'CIPROFLOXACIN'
- 'NETILMICIN'
- 'AMIKACIN'
- 'TAZOCIN'
- 'METRONIDAZOLE'
- 'VANCOMYCIN'
- 'CEFOTAXIME'
- 'AMPICILLIN'
- 'CEFUROXIME'
- 'CEFTAZIDIME'
- 'CEFTRIAXONE'
- 'PIPERACILLIN'
- 'OFLACILLIN'
- 'AZLOCILLIN'
- 'LINEZOLID'
- 'CEFALEXIN'
- 'AMOXICILLIN'
- 'MEROPENEM'
- 'IMEPENEM'
- 'IMIPENEM'

1. **ADDITIONAL DESCRIPTIVE RESULTS**

In addition to our primary late onset sepsis (LOS) definition, we report below, the risk in the matched population of:

- Exposure to bovine formula milk in the first 14 days
- NNAP-defined LOS: positive blood culture of any organism(s) from the NNAP list of “Clearly pathogenic organisms”
- NNAP-pragmatically defined late onset sepsis: positive blood culture of any organism(s) from the NNAP list of “Clearly pathogenic organisms” and “Other organisms” including CoNS [18]

|  | **Risk (%)** | | |
| --- | --- | --- | --- |
|  | **Matched population**  **(n=16,586)** | **Probiotic exposed (n=8,293)** | **Not exposed (n=8,293)** |
| Fed any bovine formula milk days 1-14 | 32.5 | 30.8 | 34.2 |
| NNAP-defined LOS | 4.4 | 4.2 | 4.5 |
| NNAP -pragmatically defined LOS | 4.8 | 4.7 | 4.9 |

**Code list for the definition of late onset sepsis in the NNRD field “PrincipalDiagnosesAtDischarge”**

| sepsis - confirmed bacterial (gram positive) |
| --- |
| sepsis / septicaemia - confirmed with +ve microbiology |
| e.coli sepsis / septicaemia |
| candida sepsis / septicaemia |
| group b streptococcal sepsis / septicaemia (gbs) |
| staphylococcal sepsis / septicaemia |
| staph. aureus sepsis / septicaemia |
| sepsis / septicaemia - specified - klebsiella sp. |
| sepsis / septicaemia - specified - enterobacter sp. |
| sepsis / septicaemia - specified - pseudomonas sp. |
| extended beta lactamase coliform infection/sepsis |
| listeria sepsis / septicaemia / disseminated |
| sepsis - confirmed bacterial (streptococci b positive) |
| sepsis - confirmed bacterial (streptococci positive) |
| streptococcal sepsis / septicaemia |
| salmonella sepsis |
| sepsis due to streptococcus |
| umbilical sepsis / septicaemia- group b streptococcus |

1. **NEC IN SUBGROUPS OF THE WHOLE COHORT DEFINED BY SEX, MATERNAL ETHNICITY AND INTRA-UTERINE GROWTH RESTRICTION.**

To facilitate analysis of subgroups in future meta-analyses, we report risk of severe NEC in subgroups of the whole cohort defined by sex, maternal ethnicity and intra-uterine growth restriction.

|  | | **Risk of severe NEC** | |
| --- | --- | --- | --- |
|  |  | **Exposed**  **(n = 12,161)** | **Not exposed**  **(n = 35,887)** |
| Sex | Female | 2.85% | 3.27% |
|  | Male | 3.62% | 4.06% |
|  | | | |
| Maternal ethnicity | White | 2.76% | 3.44% |
|  | Mixed / Multiple ethnic groups | 5.17% | 4.19% |
|  | Asian / Asian British | 3.88% | 3.85% |
|  | Black African / Black Caribbean / Black British | 3.77% | 4.84% |
|  | Other ethnic group | 6.84% | 3.66% |
|  | Missing | 3.84% | 3.86% |
|  | | | |
| Intra-uterine growth restriction | Growth restricted | 4.48% | 7.59% |
|  | Not growth restricted | 3.20% | 3.52% |

**5: DEFINITIONS OF EXPLORATORY OUTCOMES**

| **Outcome** | **Definition** |
| --- | --- |
| Severe brain injury | Uni or bilateral grade 3 or 4 intra-ventricular haemorrhage or cystic periventricular leukomalacia |
| Treated retinopathy of prematurity | Cryotherapy, laser therapy or injection of anti-vascular endothelial growth factor therapy in either or both eyes |
| Bronchopulmonary dysplasia | Any respiratory or ventilatory support or supplemental oxygen at 36 weeks postmenstrual age |
| Severe bronchopulmonary dysplasia | Ventilation via endotracheal tube or tracheostomy, and excluding non-invasive support or CPAP, at 36 weeks postmenstrual age |
| Time to full feeds | The day of life when the infant first has three consecutive days without any parenteral nutrition or intravenous fluid |

**6: TREATMENT OF MISSING DATA**

A complete case analysis will, in general, produce a biased estimate of the odds ratio and associated confidence interval for a binary exposure when missingness depends on both the outcome and exposure (14). To determine whether multiple imputation for missing data was required, we examined whether the value of any covariate or its missingness was associated with the exposure and outcome.

Three variables had rates of missingness of 5-10% (IMD quintile, gravidity and mode of delivery), and one variable, maternity ethnicity, was missing for 21.4% (10,299/48,048) of infants; all remaining variables had < 5% missing data. Missingness of ethnicity was not associated with severe NEC (χ^2^= 2.43, df=1, p=0.12), therefore risk of bias was deemed small and a complete case analysis was conducted, including an additional categorical level “missing” for maternal ethnicity.

92.0% (44,190/48,048) infants in the cohort had complete data for the variables included in the propensity score.

**Background characteristics of infants with complete covariates vs incomplete covariates**

Infants are considered to have complete covariates if data are complete for all constituents of the propensity score excluding ethnicity, since missingness in the ethnicity variable was coded as a separate level of the Maternal Ethnicity variable.

| **Outcome** | | **Incomplete cases**  **(N = 3,858)** | **Complete cases**  **(N = 44,190)** |
| --- | --- | --- | --- |
| Birth year | 2016-2019 | 2,355 (61.0%) | 26,425 (59.8%) |
|  | 2020-2022 | 1,503 (39.0%) | 17,765 (40.2%) |
| Gestational age (weeks)  Median (Q1, Q3) | | 29.6 (27.6, 31.0) | 29.4 (27.4, 30.9) |
| Birthweight (grams)  Median (Q1, Q3) | | 1,230 (934, 1,520) | 1,210 (910, 1,500) |
| Sex | Male | 2,162 (56.0%) | 24,191 (54.7%) |
|  | Female | 1,696 (44.0%) | 19,999 (45.3%) |
| Illness severity score | 0 | 1,379 (35.7%) | 18,514 (41.9%) |
|  | 1 | 1,595 (41.3%) | 19,591 (44.3%) |
|  | 2 | 325 (8.4%) | 5,056 (11.4%) |
|  | 3 | 84 (2.2%) | 1,029 (2.3%) |
|  | Missing | 475 (12.3%) | 0 (0%) |

**7: BUILDING THE PROPENSITY SCORE MODEL**

In this study we matched pairs of infants on their propensity score (a linear function of the covariates that were included in the propensity score model). Following the DAG, we would also have included neonatal unit as a highly important variable. However, probiotics are given prophylacticly and in units that use probiotics, all infants are exposed to the same intervention. Consequently it would be very difficult to find matches who were unexposed to probiotics within the same unit. Importantly, infants who are not given probiotics in a probiotic centre are likely to have other very different characteristics e.g. be sicker, less stable or on a palliative care pathway. To control for some important unit level differences we chose instead to include two variables, Level of Care (intensive care, high dependency care, special care or normal care) and hospital network, as highly important variables.

Logistic regression models were fitted with all the critically important and highly important variables plus each of the moderately important variables added individually. The model with the largest value of the chi-squared statistic was adopted if the test statistic exceeded 1.0. This cycle was repeated, adding each remaining moderately important background variable individually, until none of the chi-squared test statistics exceeded 1 or until all variables had been included in the model. We assessed the model containing the main effects for evidence of collinearity. Any variables where the variance inflation factors exceeded five were excluded. No variables were excluded due to collinearity.

Interactions between background variables were also included in the propensity score model. To identify the interactions for inclusion, we sorted the main effects included in the model by the absolute value of their t-ratios. Starting with the variable with the highest t-ratio we examined all potential interactions with that variable. Continuous variables could interact with themselves, but binary and categorical variables could not. Potential interactions were added individually to the model and the two interactions with the largest value of the chi-squared statistic, were selected if the test statistic exceeded 2.71 (implying significance at the 5% level for a two tailed test with 1 degree of freedom). This process was repeated for each of the main effects already selected for inclusion in the propensity score model.

**8: ACKNOWLEDGEMENTS**

The authors wish to thank these individuals for their assistance with this study.

**PROBIOTICS SURVEY RESPONDENTS**

**Institution Respondent**

Calderdale Royal Hospital Karin Schwarz

Chesterfield & North Derbyshire Royal Hospital Janani Devaraj

Cumberland Infirmary Yee Mon Aung

Hull Royal Infirmary Eleanor Peirce

Leicester Royal Infirmary Deepa Panjwani

Wythenshawe Hospital Abhijeet Godhamgaonkar

New Cross Hospital Helen Moore

North Devon District Hospital Tiziana Fragapane

North Manchester General Hospital Bivan Saha

Princess of Wales Hospital Abby Parish

Queen Elizabeth Hospital, King's Lynn Sally Crane

Queen Elizabeth the Queen Mother Hospital Tracey Twyman

Queens Medical Centre, Nottingham Don Sharkey

Russells Hall Hospital Lisa Gough

Sunderland Royal Hospital Chike Onwuneme

Singleton Hospital Arun Ramachandran

Southmead Hospital Paul Mannix

St Mary's Hospital, Manchester Kristin Tanney

Torbay Hospital Esther Morris

West Cumberland Hospital Yee Mon Aung

William Harvey Hospital Vimal Vasu

Yeovil District Hospital Siba Paul

**ENGLISH AND WELSH MEMBERS OF THE UK NEONATAL COLLABORATIVE**

**Institution Lead**

Airedale General Hospital Dr Matthew Babirecki

Alder Hey Dr Rebecca Kettle

Arrowe Park Hospital Dr Anand Kamalanathan

Barnet Hospital Dr Clare Cane

Barnsley District General Hospital Dr Kavi Aucharaz

Basildon Hospital Dr Rathod Poorva

Basingstoke & North Hampshire Hospital Dr Jummy Awoseyila

Bassetlaw District General Hospital Dr L M Wong

Bedford Hospital Dr Anita Mittal

Birmingham City Hospital Dr Penny Broggio

Birmingham Heartlands Hospital Dr Pinki Surana

Birmingham Women's Hospital Dr Matt Nash

Bradford Royal Infirmary Dr Sam Wallis

Broomfield Hospital, Chelmsford Dr Ahmed Hassan

Calderdale Royal Hospital Dr Karin Schwarz

Chelsea & Westminster Hospital Dr Shu-Ling Chuang

Chesterfield & North Derbyshire Royal Hospital Dr Penelope Young

Colchester General Hospital Dr Ramona Onita

Conquest Hospital Dr Mani Kandasamy

Countess of Chester Hospital Dr Stephen Brearey

Croydon University Hospital Dr Joselyn Morris

Cumberland Infirmary Dr Rachel Smith

Darent Valley Hospital Dr Bharath Gowda

Darlington Memorial Hospital Dr Mehdi Garbash

Derriford Hospital Dr Alex Allwood

Diana Princess of Wales Hospital Dr Vijaya Hebbar

Doncaster Royal Infirmary Dr Nigel Brooke

Dorset County Hospital Dr Claire Hollinsworh

East Surrey Hospital Dr Toria Klutse

Epsom General Hospital Dr Clare Sturdy

Frimley Park Hospital Dr Sathish Krishnan

Furness General Hospital Dr Maria Hadjicosta

George Eliot Hospital Dr Sabyasachi Chowdhury

Gloucester Royal Hospital Dr Shyam Bhakthavalsala

Good Hope Hospital Dr Daniel Dogar

Great Western Hospital Dr Girish Gowda

Guy's & St Thomas' Hospital Dr Karen Turnock

Harrogate District Hospital Dr Patricia Gilbertson

Hereford County Hospital Dr Cath Seagrave

Hillingdon Hospital Dr Tristan Bate

Hinchingbrooke Hospital Dr Hilary Dixon

Homerton Hospital Dr Narendra Aladangady

Hull Royal infirmary Dr Hassan Gaili

Ipswich Hospital Dr Prathiba Pai

James Cook University Hospital Dr M Lal

James Paget Hospital Dr Oluseun Tayo

Kettering General Hospital Dr Abraham Isaac

Kings College Hospital Dr Carolina Zorro

King's Mill Hospital Dr Dhaval Dave

Kingston Hospital Dr Jonathan Filkin

Lancashire Women and Newborn Centre Dr Savi Sivashankar

Leeds General Infirmary Dr Hannah Shore

Leicester General Hospital Dr Jo Behrsin

Leicester Royal Infirmary Dr Jo Behrsin

Leighton Hospital Dr Michael Grosdenier

Lincoln County Hospital Dr Ruchika Gupta

Lister Hospital Dr Ather Ahmed

Liverpool Women's Hospital Dr Nim Subhedar

Luton & Dunstable Hospital Dr Jennifer Birch

Macclesfield District General Hospital Dr Surendran Chandrasekaran

Manor Hospital (Walsall) Dr Ashok Karupaiah

Medway Maritime Hospital Dr Ghada Ramadan

Milton Keynes General Hospital Dr I Misra

Musgrove Park Hospital Dr Chris Knight

New Cross Hospital Dr Richard Heaver

Newham General Hospital Dr Mohammad Alam

Nobles Hospital Dr Prakash Thiagarajan

Norfolk & Norwich University Hospital Dr Florence Walston

North Devon District Hospital Dr Tiziana Fragapane

North Manchester General Hospital Dr Bivan Saha

North Middlesex University Hospital Dr Cheentan Singh

Northampton General Hospital Dr Nick Barnes

Northumbria Specialist Emergency Care Hospital  Dr Sangeeta Tiwary

Northwick Park Hospital Dr Richard Nicholl

Nottingham City Hospital Dr Dush Batra

Nottingham University Hospital (QMC) Dr Dush Batra

Ormskirk District General Hospital Dr Victoria Nesbitt

Oxford University Hospitals, John Radcliffe Hospital Dr Amit Gupta

Peterborough City Hospital Dr Katharine McDevitt

Pilgrim Hospital Dr Ruchika Gupta

Pinderfields General Hospital Dr David Gibson

Poole General Hospital Dr Peter Mcewan

Princess Alexandra Hospital Dr Sanath Reddy

Princess Anne Hospital Dr Mark Johnson

Princess Royal Hospital Dr Cassie Lawn

Princess Royal Hospital Telford Dr Alison Belfitt and Dr Jennifer Brindley

Princess Royal University Hospital Dr Rashmi Gandhi

Queen Alexandra Hospital Dr Charlotte Groves

Queen Charlotte's Hospital Dr Aniko Deierl

Queen Elizabeth Hospital, Gateshead Dr Shilpa Ramesh

Queen Elizabeth Hospital, King's Lynn Dr Salamatu Jalloh

Queen Elizabeth Hospital, Woolwich Dr Julia Croft

Queen Elizabeth the Queen Mother Hospital Dr Bushra Abdul-Malik

Queen's Hospital, Burton on Trent Dr Dominic Muogbo

Queen's Hospital, Romford Dr Ambalika Das

Queen's Hospital, Romford 2 Dr Khalid Mannan

Rosie Maternity Hospital, Addenbrookes Dr Shazia Hoodbhoy

Rotherham District General Hospital Dr Soma Sengupta

Royal Albert Edward Infirmary Dr Christos Zipitis

Royal Berkshire Hospital Dr Kemy Naidoo

Royal Bolton Hospital Dr Dinakar Seshadri

Royal Cornwall Hospital Dr Chris Warren

Royal Derby Hospital Dr Nigel Ruggins

Royal Devon & Exeter Hospital Dr Chrissie Oliver

Royal Hampshire County Hospital Dr Lucinda Winckworth

Royal Lancaster Infirmary Dr Joanne Fedee

Royal Oldham Hospital Dr Anitha Vayalakkad

Royal Preston Hospital Dr Richa Gupta

Royal Stoke University Hospital Dr Julia Uffindell

Royal Surrey County Hospital Dr Jo MacLeod

Royal Sussex County Hospital Dr Cassie Lawn

Royal United Hospital Dr Rebecca Winterson

Royal Victoria Infirmary Dr Naveen Athiraman

Russells Hall Hospital Dr Muhammad Khurshid

Salisbury District Hospital Dr Jim Baird

Scarborough General Hospital Dr Adedayo Owoeye

Scunthorpe General Hospital Dr Umapathee Majuran

Sheffield Children's Hospital Dr Richard Lindley

Southend Hospital Dr Vineet Gupta

Southmead Hospital Dr Faith Emery and Dr Madhavi Parvathareddy

St George's Hospital Dr Donovan Duffy

St Helier Hospital Dr Salim Yasin

St James University Hospital Dr Hannah Shore

St Mary's Hospital, IOW Dr Akinsola Ogundiya

St Mary's Hospital, London Dr Aniko Deierl

St Mary's Hospital, Manchester Dr Arin Mukherjee

St Michael's Hospital Dr Pamela Cairns

St Peter's Hospital Dr Vennila Ponnusamy

St Richard's Hospital Dr Victoria Sharp

Stepping Hill Hospital Dr Carrie Heal

Stoke Mandeville Hospital Dr Sanjay Salgia

Sunderland Royal Hospital Dr Imran Ahmed

Tameside General Hospital Dr Helen Purves

The Jessop Wing, Sheffield Dr Porus Bastani

The Royal Free Hospital Dr Eleanor Bond

The Royal London Hospital - Constance Green Dr Divyen Shah

Torbay Hospital Dr Esther Morris

Tunbridge Wells Hospital  Dr Mithun Urs

University College Hospital Dr Giles Kendall

University Hospital Coventry Dr Puneet Nath

University Hospital Lewisham Dr Igor Fierens

University Hospital of North Durham Dr Mehdi Garbash

University Hospital of North Tees Dr Hari Kumar

Victoria Hospital, Blackpool Dr Peter Curtis

Warrington Hospital Dr Delyth Webb

Warwick Hospital Dr Sumedha Bird

Watford General Hospital Dr Sankara Narayanan

West Cumberland Hospital Dr Yee Mon Aung

West Middlesex University Hospital Dr Elizabeth Eyre

West Suffolk Hospital Dr Tayyaba Aamir

Wexham Park Hospital Dr Angela Yannoulias

Whipps Cross University Hospital Dr Caroline Sullivan

Whiston Hospital Dr Ros Garr

Whittington Hospital Dr Wynne Leith

William Harvey Hospital Dr Shaveta Mulla

Worcestershire Royal Hospital Dr Anna Gregory

Worthing Hospital Dr Edward Yates

Wythenshawe Hospital  Dr Abijeet Godhamgaonkar

Yeovil District Hospital Dr Siba Paul

York District Hospital Dr Sundeep Sandhu

Singleton Hospital Dr Arun Ramachandran

Princess of Wales Hospital Dr Abby Parish

The Grange University Hospital Dr Anitha James

Glan Clwyd Hospital Dr Ambrose Onibere

Wrexham Maelor Hospital Dr Artur Abelian

Ysbyty Gwynedd Dr Shakir Saeed

University Hospital of Wales Dr Nitin Goel

Prince Charles Hospital Dr Shikha Jain

Glangwili General Hospital Dr Prem Pitchaikani

**FIGURE S1: DIRECTED ACYCLIC GRAPH (DAG) SUMMARISING CRITICAL RELATIONSHIPS INVOLVED IN THE ASSOCIATION BETWEEN PROBIOTICS AND SEVERE NEC.**

**
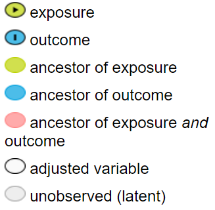

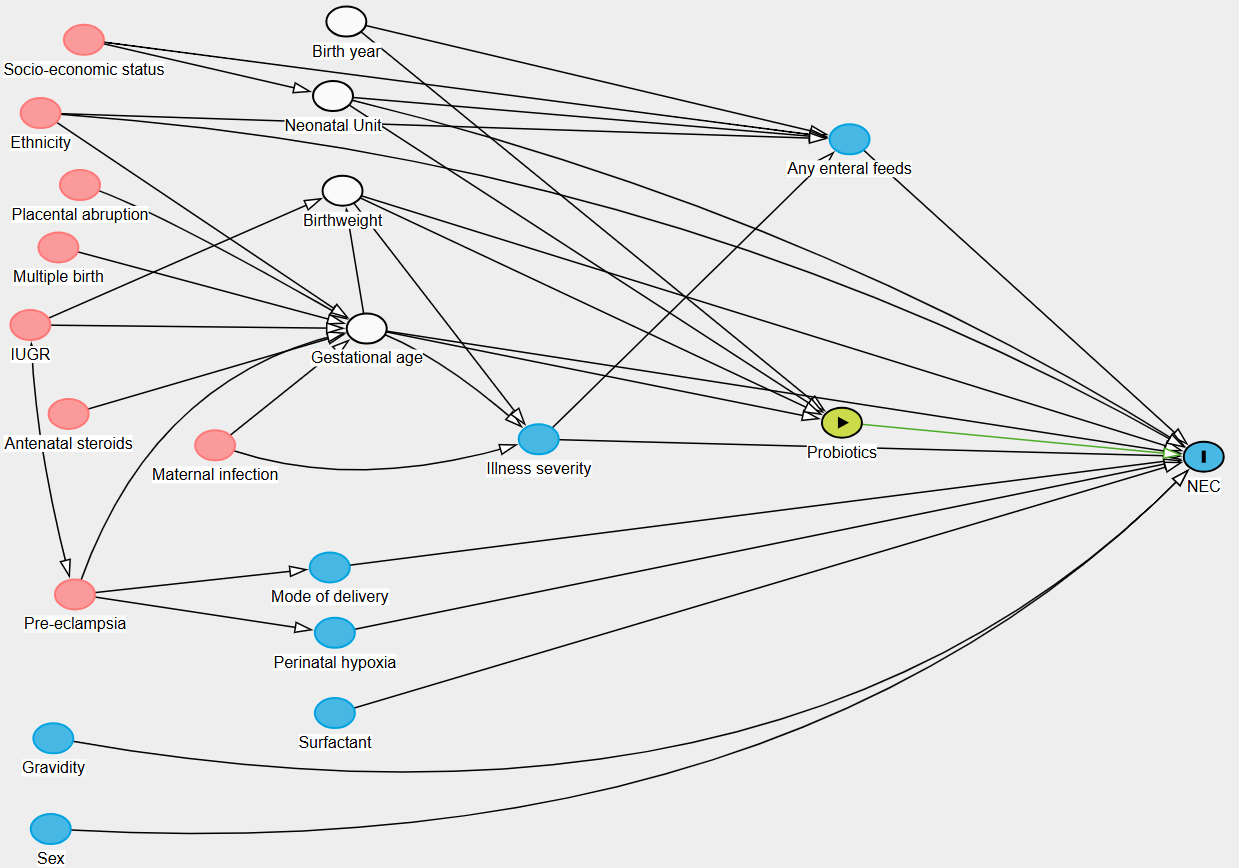
**

**FIGURE S2: BALANCE PLOT SHOWING STANDARDISED DIFFERENCES BETWEEN PROBIOTIC GROUPS BEFORE AND AFTER PROPENSITY MATCHING**

**
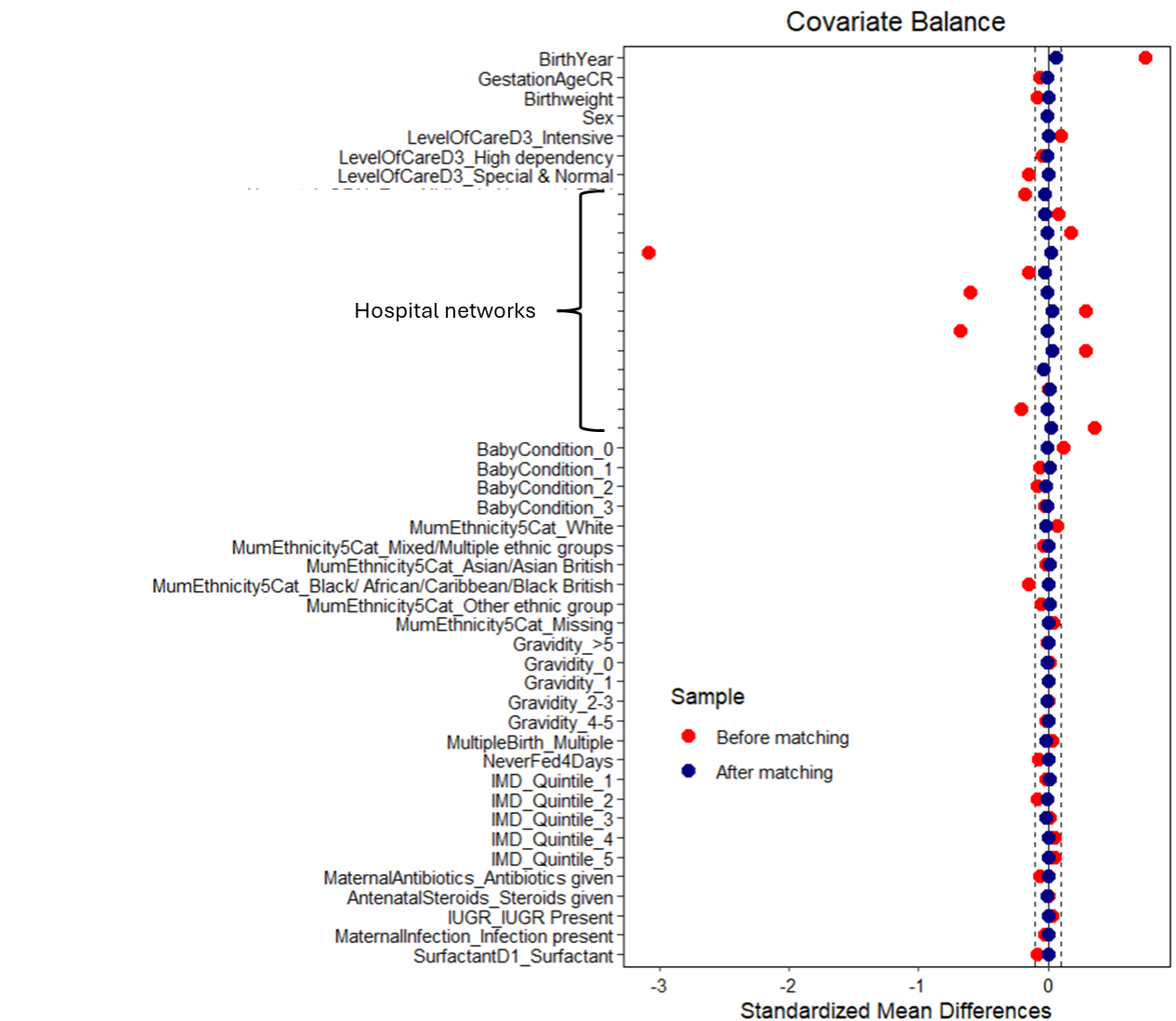
**

**FIGURE S3: DISTRIBUTION OF PROPENSITY SCORES**

The figure below shows the distribution of propensity scores for four groups of infants, those included in and excluded from the matched population in the exposed and unexposed groups.


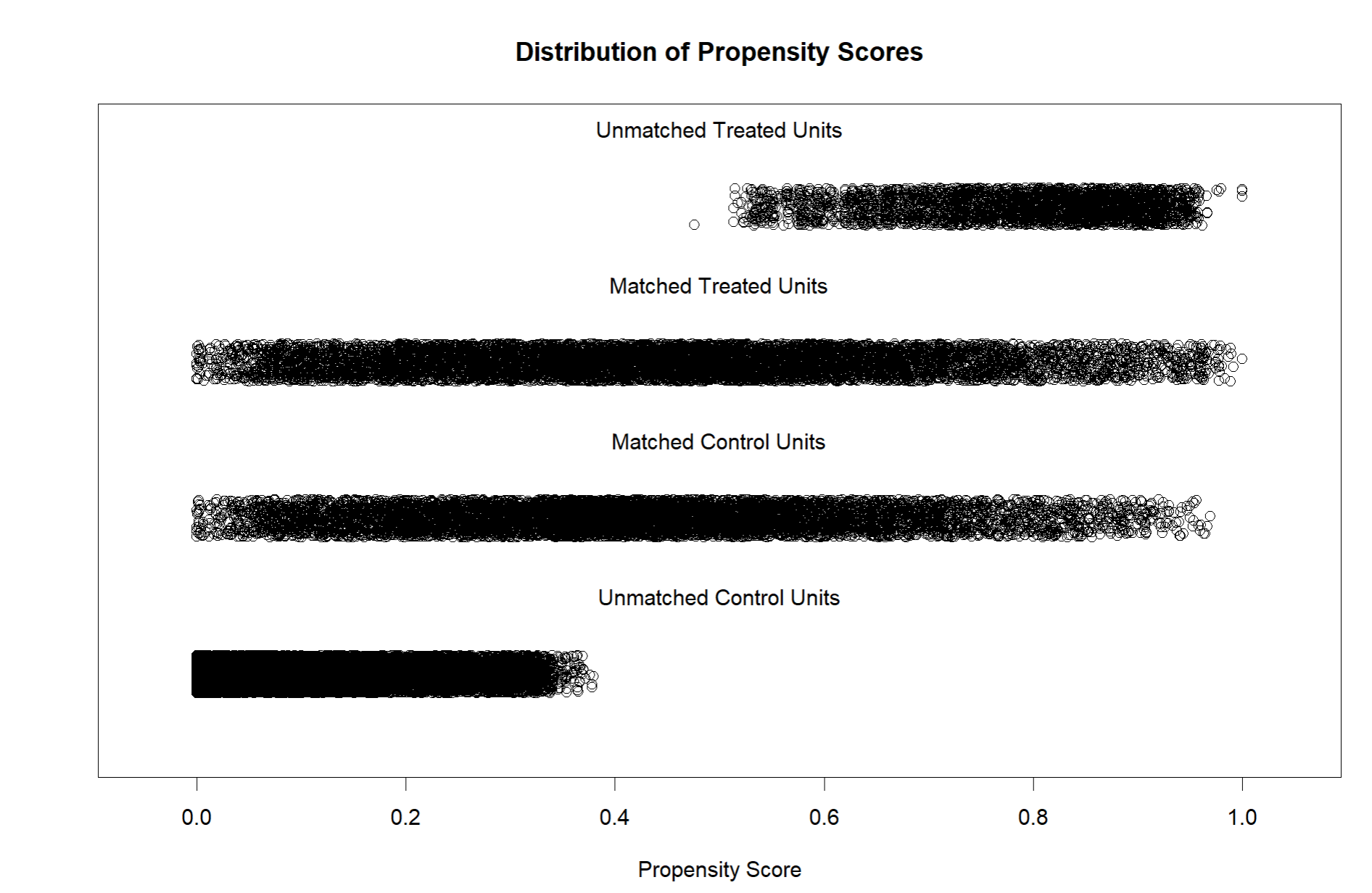


**FIGURE S4: KAPLAN MEIER PLOT OF SURVIVAL BY PROBIOTIC EXPOSURE**


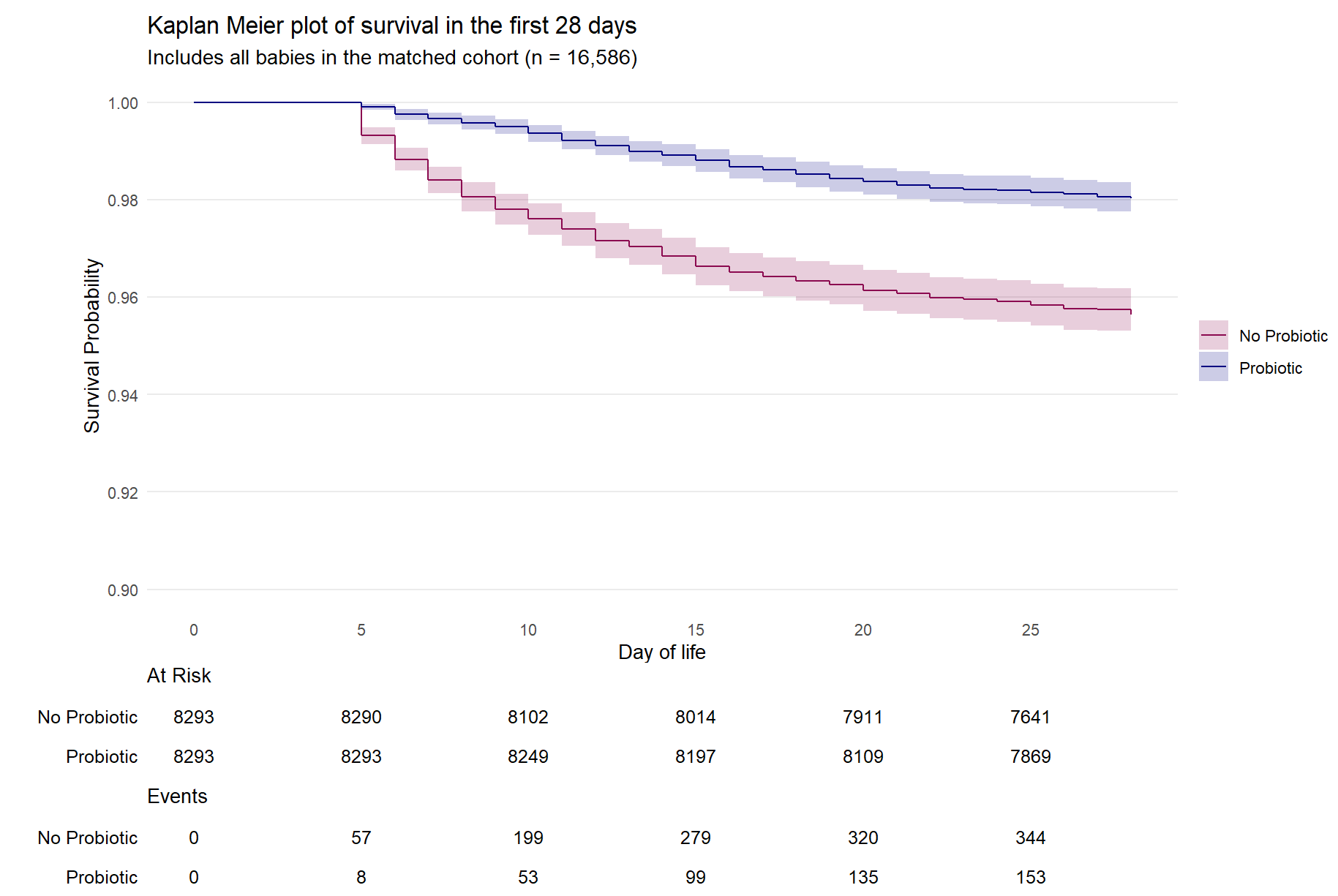


**TABLE S1: PROBIOTICS PRODUCTS USED DURING THE STUDY PERIOD AS EXTRACTED FROM THE NNRD DAILY DRUGS FIELD**

| **Product Name** | **Probiotic Strains** |
| --- | --- |
| Labinic and LB2 | *Lactobacillus acidophilus, Bifidobacterum bifidum* and *Bifidobacterium infantis* |
| Proprems | *Bifidobacterium infantis Bb-02 (DSM 33361), Bifidobacterium lactis (BB-12®)* and *Streptococcus thermophilus (TH-4®)* |
| Bio-kult | *Lactobacillus paracasei PXN® 37™, Lactobacillus rhamnosus PXN® 54™, Streptococcus thermophilus PXN® 66™, Lactobacillus helveticus PXN® 35™, Bifidobacterium breve PXN® 25™, Bifidobacterium infantis PXN® 27™* and *Lactobacillus delbrueckii ssp. bulgaricus PXN® 39™* |
| Infloran | *Bifidobacterium bifidum NCDO 2203* and *Lactobacillus acidophilus NCDO 1784* |

**TABLE S2: CONCEPTS FROM THE DAG AND THE NNRD VARIABLES USED TO OPERATIONALISE THOSE CONCEPTS**

| **Concept from DAG** | **Class within DAG** | **NNRD field(s) used to operationalise the concept** | **Rank and mechanism in propensity score** |
| --- | --- | --- | --- |
| Probiotics | Exposure | DrugsDay 0-14 days  Any documented probiotic: Labinic, Proprems, Bifidobacterium, Bio-kult, Infloran, LB2 | Binary (yes, no) |
| Severe necrotising enterocolitis | Outcome | Combination fields (Supplementary materials 2) | Binary (yes, no) |
| Gestational age | Adjusted variable | GestationWeeks and GestationDays | Critical (binary < 28 weeks or ≥  28 weeks)  AND  High (continuous, days) |
| Birth year | Adjusted variable | BirthYear | Critical (categorical: 2016-2018, 2019-2020 or 2021-2022) AND  High (categorical, years) |
| Birthweight | Adjusted variable | Birthweight | High (continuous, grams) |
| Neonatal Unit | Adjusted variable | ProviderNHSCode (hospital network on day 3) | High (categorical: 13 Operational Delivery Network) |
|  |  | BAPM2011 (maximum level first 4 days) | High (categorical: intensive, high dependency, special, normal ) |
| Sex | Adjusted variable | SexPhenotype | High (Binary: Male, Female) |
| Maternal ethnicity | Adjusted variable | MumEthnicity | Moderate (categorical: White, Mixed, Asian/Asian British, Black/Black British, Other, Missing.) |
| Index of Multiple Deprivation | Adjusted variable | PostCodeMotherLSOA (Lower Level Super Output Area) | Moderate (categorical: 5 quintiles) |
| Gravidity | Adjusted variable | NumberOfPreviousPregnancies | Moderate (categorical: 0, 1, 2-3, 4-5, >5) |
| Multiple birth | Adjusted variable | FetusNumber | Moderate (binary: multiple, singleton) |
| Maternal infection | Adjusted variable | MaternalPyrexiaInLabour38c OR IntrapartumAntibioticsGiven OR Chorioamnionitis. | Moderate (binary: infection, no infection) |
| Antenatal steroids | Adjusted variable | SteroidsAntenatalGiven | Moderate (binary: any, none) |
| Illness severity score^1^ | Adjusted variable | InotropesGiven, DrugsDay, RespiratorySupport, NitricOxide  Day 1 or 2, score 1 for any inotrope, invasive respiratory support and nitric oxide. | Moderate (categorical 0,1,2,3) |
| Intrauterine growth restriction | Adjusted variable | BW_UKWHO (birthweight-for-age z-score < -2 [24]) | Moderate (binary: yes,no) |
| Surfactant | Adjusted variable | SurfactantGivenResuscitation, DrugsDaily (day of birth only) | Moderate (binary: yes,no) |
| Enteral feeds | Adjusted variable | DayEnteralFeeds  (Day one to four) | Moderate (binary: yes, no) |
| Gut ischaemia | Unobserved | Not applicable | Not recorded in NNRD |
| Pre-eclampsia^2^ | Ancestor | ProblemsDuringPregnancy | Moderate (binary: yes,no) |
| Placental abruption^2^ | Ancestor | ProblemsDuringPregnancy | Moderate (binary: yes,no) |
| Mode of delivery^2^ | Ancestor | ModeOfDelivery | Moderate (categorical: vaginal, caesarean, missing) |
| Perinatal hypoxia^2^ | Ancestor of outcome | MethodsOfResuscitation  (presence of any of cardiac compressions or any drug) | Moderate (binary: yes,no) |

^1^ Illness severity score in the first two days of life. Receiving any one of inotropes, invasive respiratory support or nitric oxide, in the first two days of life, added one to the illness severity score. Illness severity was therefore scored from zero to three.

^2^These variables were not included in the propensity score because the chi-squared statistics did not exceed one during the model building process

**TABLE S3: NUMBER OF BABIES CONTRIBUTED BY EACH NEONATAL UNIT**

24 units contributed fewer than 10 infants to the cohort. To facilitate anonymity those units have been removed from the table below. In the case of units who contributed fewer than 10 infants to the matched population, the number of infants in the matched cohort has been suppressed in the table below.

| **Unit ID** | **Total  infants** | **Matched  population  infants** |  | **Unit ID** | **Total  infants** | **Matched  population  infants** |  | **Unit ID** | **Total  infants** | **Matched  population  infants** |
| --- | --- | --- | --- | --- | --- | --- | --- | --- | --- | --- |
| 1 | 1111 | 811 |  | 36 | 509 | 93 |  | 71 | 224 | 66 |
| 2 | 1012 | 132 |  | 37 | 501 | 140 |  | 72 | 224 | 42 |
| 3 | 935 | 522 |  | 38 | 497 | 53 |  | 73 | 224 | <10 |
| 4 | 889 | 245 |  | 39 | 485 | 324 |  | 74 | 222 | 97 |
| 5 | 879 | 489 |  | 40 | 465 | 378 |  | 75 | 220 | 149 |
| 6 | 868 | 250 |  | 41 | 450 | 90 |  | 76 | 218 | <10 |
| 7 | 847 | 161 |  | 42 | 430 | 21 |  | 77 | 218 | 77 |
| 8 | 808 | 479 |  | 43 | 429 | 97 |  | 78 | 213 | 106 |
| 9 | 782 | 208 |  | 44 | 408 | 45 |  | 79 | 207 | 55 |
| 10 | 776 | 382 |  | 45 | 401 | 236 |  | 80 | 207 | 125 |
| 11 | 756 | <10 |  | 46 | 385 | <10 |  | 81 | 205 | <10 |
| 12 | 750 | 501 |  | 47 | 384 | <10 |  | 82 | 202 | <10 |
| 13 | 740 | 359 |  | 48 | 379 | <10 |  | 83 | 200 | 125 |
| 14 | 723 | 555 |  | 49 | 377 | <10 |  | 84 | 199 | 35 |
| 15 | 716 | 465 |  | 50 | 362 | 204 |  | 85 | 198 | 81 |
| 16 | 709 | 171 |  | 51 | 347 | 87 |  | 86 | 190 | <10 |
| 17 | 693 | 14 |  | 52 | 339 | <10 |  | 87 | 189 | 87 |
| 18 | 668 | 117 |  | 53 | 314 | <10 |  | 88 | 187 | 12 |
| 19 | 668 | 53 |  | 54 | 304 | <10 |  | 89 | 181 | 32 |
| 20 | 665 | 142 |  | 55 | 304 | 60 |  | 90 | 181 | 35 |
| 21 | 659 | <10 |  | 56 | 300 | 122 |  | 91 | 181 | 42 |
| 22 | 657 | 369 |  | 57 | 282 | 63 |  | 92 | 179 | 87 |
| 23 | 654 | 223 |  | 58 | 279 | 123 |  | 93 | 178 | 97 |
| 24 | 618 | <10 |  | 59 | 267 | 126 |  | 94 | 177 | 77 |
| 25 | 615 | <10 |  | 60 | 264 | 38 |  | 95 | 174 | 14 |
| 26 | 614 | 379 |  | 61 | 253 | 149 |  | 96 | 171 | 81 |
| 27 | 589 | 480 |  | 62 | 251 | 143 |  | 97 | 171 | 91 |
| 28 | 586 | 115 |  | 63 | 250 | 51 |  | 98 | 169 | 124 |
| 29 | 583 | 136 |  | 64 | 249 | 164 |  | 99 | 167 | 100 |
| 30 | 575 | 278 |  | 65 | 242 | 141 |  | 100 | 165 | 16 |
| 31 | 551 | 60 |  | 66 | 233 | 223 |  | 101 | 165 | 105 |
| 32 | 540 | 30 |  | 67 | 233 | 199 |  | 102 | 162 | 128 |
| 33 | 539 | 249 |  | 68 | 227 | 110 |  | 103 | 162 | 80 |
| 34 | 536 | 300 |  | 69 | 226 | 50 |  | 104 | 159 | <10 |
| 35 | 526 | 372 |  | 70 | 224 | <10 |  | 105 | 159 | 144 |

| **Unit ID** | **Total  infants** | **Matched  population  infants** |  | **Unit ID** | **Total  infants** | **Matched  population  infants** |
| --- | --- | --- | --- | --- | --- | --- |
| 106 | 159 | 89 |  | 143 | 41 | 17 |
| 107 | 159 | 120 |  | 144 | 40 | 10 |
| 108 | 158 | 69 |  | 145 | 39 | 27 |
| 109 | 156 | 62 |  | 146 | 38 | <10 |
| 110 | 150 | 27 |  | 147 | 36 | <10 |
| 111 | 147 | 79 |  | 148 | 35 | <10 |
| 112 | 144 | 66 |  | 149 | 29 | 21 |
| 113 | 143 | <10 |  | 150 | 27 | <10 |
| 114 | 141 | 18 |  | 151 | 26 | <10 |
| 115 | 141 | 113 |  | 152 | 25 | <10 |
| 116 | 139 | 80 |  | 153 | 24 | 11 |
| 117 | 136 | 14 |  | 154 | 23 | 11 |
| 118 | 136 | <10 |  | 155 | 22 | 17 |
| 119 | 131 | 16 |  | 156 | 22 | <10 |
| 120 | 127 | 69 |  | 157 | 20 | <10 |
| 121 | 119 | <10 |  | 158 | 20 | <10 |
| 122 | 113 | 13 |  | 159 | 19 | <10 |
| 123 | 96 | 26 |  | 160 | 18 | <10 |
| 124 | 96 | 25 |  | 161 | 18 | <10 |
| 125 | 94 | 10 |  | 162 | 18 | 12 |
| 126 | 91 | 80 |  | 163 | 17 | <10 |
| 127 | 90 | 14 |  | 164 | 17 | <10 |
| 128 | 89 | 10 |  | 165 | 16 | <10 |
| 129 | 85 | 30 |  | 166 | 16 | <10 |
| 130 | 84 | 62 |  | 167 | 16 | 10 |
| 131 | 83 | <10 |  | 168 | 16 | <10 |
| 132 | 77 | 49 |  | 169 | 16 | 13 |
| 133 | 74 | 44 |  | 170 | 15 | 13 |
| 134 | 73 | 22 |  | 171 | 15 | <10 |
| 135 | 70 | 34 |  | 172 | 15 | <10 |
| 136 | 60 | <10 |  | 173 | 15 | <10 |
| 137 | 55 | 26 |  | 174 | 15 | 14 |
| 138 | 50 | <10 |  | 175 | 13 | <10 |
| 139 | 46 | <10 |  | 176 | 12 | 11 |
| 140 | 44 | 16 |  | 177 | 11 | <10 |
| 141 | 44 | 19 |  | 178 | 11 | 10 |
| 142 | 42 | 23 |  | 179 | 10 | <10 |

**TABLE S4: RESULTS OF THE ANALYSIS OF THE FULL COHORT (n=48,048)**

| **Outcome** | **Exposed group**  **(n=12,161)**  **Risk (%) (n)** | **Unexposed group**  **(n=35,887)**  **Risk (%) (n)** | **Adjusted odds ratio^1^** | **95% CI** |
| --- | --- | --- | --- | --- |
| **Severe NEC** | 3.3 (398) | 3.7 (1,330) | 0.82 | 0.67-0.99 |
| **NNAP defined NEC** | 4.5 (521) | 5.7 (1,766) | 0.82 | 0.71-0.94 |
| **Pragmatically defined NEC** | 6.5 (778) | 7.6 (2,697) | 0.85 | 0.73-1.00 |
| **Late onset sepsis** | 4.4 (541) | 4.5 (1,616) | 0.96 | 0.87-1.05 |
| **Survival to discharge** | 96.5 (11,739) | 94.8 (34,019) | 1.64 | 1.30-2.08 |

^1^ Adjusted for: birth year, hospital network, mother’s ethnicity, IMD quintile of mother’s residential address, whether the mother received antenatal steroids, whether the mother experienced an infection around the time of the birth, maternal gravidity, whether or not this was a multiple birth, gestational age, birthweight, sex, intrauterine growth restriction, the infant’s severity of illness in the first two days of life, whether the infant received surfactant on day 1, whether the infant received any enteral feeds in the first 4 days, highest level of care in the first 4 days of life.
